# Role of MicroRNAs in Acute Myeloid Leukemia

**DOI:** 10.3390/genes16040446

**Published:** 2025-04-11

**Authors:** Aneta Wiśnik, Dariusz Jarych, Kinga Krawiec, Piotr Strzałka, Natalia Potocka, Magdalena Czemerska, Aleksandra Sałagacka-Kubiak, Agnieszka Pluta, Agnieszka Wierzbowska, Izabela Zawlik

**Affiliations:** 1 Department of Hematology, Medical University of Lodz, 93-510 Lodz, Poland; 2 Copernicus Memorial Multi-Specialist Oncology and Trauma Center, 93-510 Lodz, Poland; 3 Laboratory of Virology, Institute of Medical Biology, Polish Academy of Sciences, 93-232 Lodz, Poland; 4 Laboratory of Molecular Biology, Centre for Innovative Research in Medical and Natural Sciences, Collegium Medicum, University of Rzeszow, 35-959 Rzeszow, Poland; 5 Department of General Genetics, Faculty of Medicine, Collegium Medicum, University of Rzeszow, 35-959 Rzeszow, Poland

**Keywords:** microRNA, acute myeloid leukemia, non-coding RNA

## Abstract

MicroRNA (miRNA), a significant class of regulatory non-coding RNA (ncRNA), can regulate the expression of numerous protein-coding messenger RNAs (mRNAs). miRNA plays an important part in shaping the human transcriptome. So far, in the human genome, about 2500 miRNAs have been found. Acute myeloid leukemia (AML) belongs to a malignant clonal disorder of hematopoietic stem cells and is characterized by the uncontrolled clonal proliferation of abnormal progenitor cells in the bone marrow and blood. For the past several years, significant scientific attention has been attracted to the role of miRNAs in AML, since alterations in the expression levels of miRNAs may contribute to AML development. This review describes the main functions of non-coding RNA classes and presents miRNA biogenesis. This study aims to review recent reports about altered microRNA expression and their influence on AML cell survival, cell cycle, and apoptotic potential. Additionally, it summarizes the correlations between miRNAs and their target mRNAs in AML and outlines the role of particular miRNAs in AML subtypes according to ELN recommendations.

## 1. Introduction

MicroRNAs (miRNAs), one of the ncRNA classes, are short molecules, typically composed of 19–25 nucleotides, which can regulate gene expression by reducing target genes’ expression (either by degrading mRNA or inhibiting mRNA translation) [1]. miRNA can function as oncogenes (oncomiRs) or tumor suppressors. Due to their high stability in body fluids and tissues, miRNAs are promising biomarkers of various diseases, including cancers [2,3]. miRNA levels can be assessed by measuring their expression. The rapid development of molecular techniques and advancements in transcriptomics have expanded our understanding of miRNA and gene expression in oncology [4]. miRNA biogenesis is complex and involves several stages, beginning with transcription of DNA sequences to produce primary miRNA transcripts (pri-miRNAs). These are then processed into precursor miRNAs (pre-miRNAs) and, ultimately, into mature miRNAs [5,6]. Dysregulations of expression levels of various miRNAs are described in multiple diseases, including AML. Abnormal miRNA expression levels may serve as a valuable diagnostic or prognostic marker in AML [7]. In this review, we describe the different classes of ncRNA, summarize miRNA biogenesis, and examine alterations in miRNA expression in the context of AML.

## 2. Types of Non-Coding RNAs

It has been confirmed that less than 2% of the human genome undergoes the transcription and translation processes that lead to protein synthesis, while the rest consists of non-coding DNA. The ENCODE and FANTOM projects have shown that most non-coding DNA sequences are transcriptionally active. The transcribed but not translated sequences are known as non-coding RNAs (ncRNAs) [8,9,10,11]. ncRNA includes rRNAs (ribosomal RNAs), tRNAs (transfer RNAs), siRNAs (small interfering RNAs), miRNAs (microRNAs), lncRNAs (long non-coding RNAs), circRNAs (circular RNAs), piRNAs (piwi-interacting RNAs), snRNAs (small nuclear RNAs), snoRNAs (small nucleolar RNAs), eRNA (enhancer-derived RNA), paRNA (promoter-associated RNA), and others. In eukaryotic cells, we can distinguish regulatory and housekeeping types of ncRNA. The first group of ncRNAs plays an epigenetic function and regulates gene expression at the post-transcriptional and transcriptional stages, while the second group controls essential cellular functions. Structurally, non-coding RNAs are divided into short (fewer than 200 nucleotides) and long (more than 200 nucleotides) categories [12,13]. ncRNAs play a pivotal role in many biological processes, including translation (e.g., rRNA, tRNA, snoRNA, snRNA), control of epigenetic variations (e.g., lncRNA), alternative splicing (e.g., snRNA, snoRNA), gene expression regulation (e.g., miRNA, eRNA, snRNA, lncRNA, siRNA, piRNA, paRNA), and miRNA regulation (e.g., circRNA). The primary function of miRNAs is to regulate gene expression at the post-transcriptional level by destabilizing mRNA or inhibiting translation. miRNA targets over 60% of protein-coding genes, which can be responsible for essential cellular processes such as cell growth, apoptosis, differentiation, development, or the development of the disease process [14]. Over the past few decades, advancements in high-throughput molecular biology techniques, particularly those based on sequencing and complementary hybridization, have greatly expanded the amount of data available on ncRNAs. However, despite the growth in our knowledge regarding ncRNA, the functions of many remain unknown [15,16]. Our present understanding of ncRNAs and the genes they regulate is summarized in Table 1 below.

## 3. MicroRNA Biogenesis

MicroRNAs are small, endogenous, single-stranded, approximately 19–25 nucleotides in length, highly evolutionarily conserved molecules, and their roles continue to be explored. These short regulatory RNAs mediate post-transcriptional silencing of genes, typically by complementary binding to target mRNAs in the location of their 3′ untranslated regions (UTRs) [39,40]. One miRNA has the potential to control the expression of numerous messenger RNAs (mRNAs), resulting in mRNAs’ translational repression or degradation [41]. miRNAs play a role in regulating numerous cellular physiological processes, and alterations in their expression level can significantly influence the progression of numerous diseases [42].

The first miRNA, lin-4, was discovered in the nematode Caenorhabditis elegans in 1993. It was shown to be essential for postembryonic development by downregulating the expression of the protein LIN-14 [43].

In the human genome, two categories of miRNA are distinguished: intragenic and intergenic. These two groups differ in the transcription process and location in the genome. Intragenic miRNA is transcribed by RNA polymerase II (Pol II) and intergenic by RNA polymerase II or III (Pol III). In a significant proportion, the first group is transcribed together with their host genes by the same promoters, while the other group, using their own promoters, can be transcribed autonomously from host genes or co-transcribed with neighboring genes. Emerging studies have revealed that this co-transcription is not an absolute rule in the case of intragenic miRNAs. These two categories of miRNA are located differently in the genome—intragenic miRNA within protein-coding and non-coding genes, and intergenic between genes. Intragenic miRNAs can be further classified based on their position into intronic, exonic, exon-intron junction, and antisense gene strands [44]. Sometimes, miRNA genes are organized into clusters, where multiple miRNA genes are present at the same locus and can be transcribed simultaneously into one long primary transcript that encodes several miRNAs [45].

The production of miRNAs is a complicated process that involves several stages. Depending on the origin of the miRNA, its biogenesis is divided into two pathways: canonical and non-canonical. This study will focus on the canonical pathway, which is the most prevalent route of miRNA synthesis. Initially, DNA sequences are transcribed into primary miRNA transcripts (pri-miRNAs). These pri-miRNAs are then processed into precursor miRNAs (pre-miRNAs) and, ultimately, into mature miRNAs [46,47].

The canonical pathway starts with the transcription of miRNA genes into pri-miRNA by Pol II or III. These are long stem-loop structures composed of hundreds to thousands of nucleotides (nts), and the pri-miRNA carries the mature miRNA within the 3′ or 5′ arm (or both) [48,49]. The pri-miRNA undergoes splicing similarly to protein-coding mRNAs, and during polyadenylation, it acquires a 5′ m7G cap and a 3′ poly(A) tail [50]. Then, the pri-miRNA undergoes cleavage by the Microprocessor, a complex that includes one Drosha ribonuclease III (DROSHA) molecule and two molecules of its cofactor DiGeorge syndrome critical region 8 (DGCR8), a dsRNA binding protein. Microprocessor has a heterotrimeric structure. DROSHA cleaves approximately 11 base pairs from the ssRNA-dsRNA junction, which DGCR8 recognizes. Consequently, a smaller stem-loop precursor miRNA (pre-miRNA) is realized, about 80 nucleotides long, with one defined mature miRNA end [51,52,53]. The GHG motif in a stem part of pri-miRNA supports DROSHA in precisely recognizing the cleavage site [54].

The pre-miRNA is then transported to the cytoplasm by the RanGTP/Exportin-5 complex, which prevents nuclear degradation and enables transport [55]. RanGTP is subsequently hydrolyzed to RanGDP [56]. The pre-miRNA undergoes further processing in the cytoplasm by a complex which cleaves its loop. As a consequence, a 22-nt double-stranded miRNA duplex is realized. Duplexes have 2-nt 3′ overhangs at both ends, providing the second end of mature miRNA. This cleaving complex is composed of cytoplasmic ribonuclease III Dicer, transactivation response element RNA-binding protein (TRBP), and a protein activator of the double-stranded RNA-activated protein kinase (PACT) [57,58]. The duplex miRNA/*miRNA consists of the mature miRNA and the passenger strand (denoted by a star*) [59]. The duplex interacts with an Argonaut protein (AGO), and the duplex unwinds, leading to the destruction of the star strand. Along the course of one of the processes, when the central region of the duplex is complimentary, the star strand is cleaved by Argonaute 2 (AGO2), which is further degraded by the C3PO nuclease complex. The mature miRNA binds to the AGO protein, forming a miRNA-induced silencing complex (miRISC). It is possible that both strands of the precursor are incorporated into the RISC complex, with both strands serving as sources for mature miRNAs. When the mature miRNA derives from the 5′ strand of the stem-loop precursor, it is referred to as 5p, and when it derives from the 3′ strand, it is called 3p [60,61]. miRNA generally exhibit a complementarity with the 3′ UTR of their target mRNAs, through a crucial segment known as the “seed” region. This specific sequence, situated at the 2–7 nucleotide positions of the 5′ end of the mature miRNA, plays a vital role in the binding process. However, interactions with other regions, including the 5′ UTR, gene promoters, or coding sequences, have also been reported [4,62,63,64].

A non-canonical miRNA biogenesis pathway involves miRtrons, a class of miRNAs located within the introns of coding genes. MiRtrons form hairpin-containing lariat structures, which, after splicing, bypass Microprocessor cleavage. These molecules are debranched by the debranching enzyme 1 (DRB1) and are subsequently treated as pre-miRNA, ready for exporting to the cytoplasm by Exportin-5 [55].

**Figure 1:**
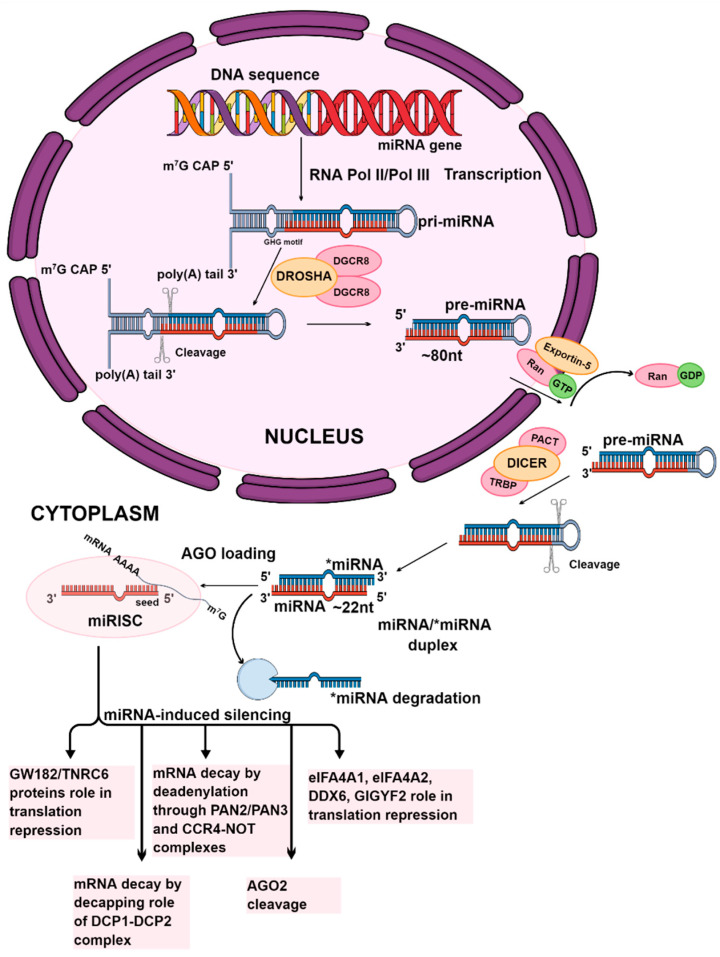
The canonical pathway of miRNA biogenesis. The initial stages of miRNA synthesis occur in the nucleus. The first stage is the transcription of the miRNA gene by RNA polymerase II or III. This process produces the primary miRNA transcript. The Microprocessor complex, consisting of one Drosha molecule and two DGCR8 molecules, cleaves the pri-miRNA to form precursor miRNA. The precursor miRNA is then transported to the cytoplasm by the RanGTP/Exportin-5 complex. In the cytoplasm, the Dicer enzyme, with TRBP and PACT, cut the pre-miRNA, which generates miRNA/*miRNA duplexes. Mature miRNA, which is defined in one strand of the duplex, after its unwinding, as a single-stranded particle, is preserved within an active RISC complex, leading to the formation of miRISC, while the passenger strand is degraded. Subsequently, the expression of target mRNA is silenced by either cleaving the mRNA or inhibiting translation. The graphic was created using the program Mind the Graph (mindthegraph.com).

After miRISC connects with its target, it triggers either mRNA decay or translation repression. The miRISC complex requires miRNA and Argonaute proteins (specifically AGO1-4 in humans) to become functional. However, it has been proved that miRISC consists of many other components: GW182 proteins (a family of proteins rich in tryptophan and glycine repeats, specifically TNRC6A-C in humans), the CCR4-NOT complex (carbon catabolite repression—negative on TATA-less complex), the PAN2/PAN3 complex (two subunits of the poly(A)-specific ribonuclease PAN complex), DCP1 (decapping protein 1), DDX6 (RNA helicase), and GIGYF2 (GRB10 interacting GYF protein 2). Several mechanisms of miRNA-induced silencing exist. GW182 proteins have been shown to be involved in the structural formation of the miRISC complex and in repressing target mRNA. They interact with AGO proteins through their N-terminal domain, while their C-terminal silencing domain is responsible for either transcriptional repression or mRNA degradation. Although their full function remains unclear, it has been observed that these proteins can silence the expression of target mRNAs independently of AGO proteins. Translation repression also occurs during the deadenylation process, which is mediated by the deadenylase CCR4-NOT complex. Additionally, the PAN2/PAN3 complex contributes to miRNA-induced deadenylation. In addition, the mechanism of translational repression entails decapping, which refers to the removal of the 5′ cap structure from mRNA by the decapping enzyme DCP2, activated by DCP1. The recruitment of DDX6 and GIGYF2 can also lead to transcriptional repression by inhibiting the eIF4F (eukaryotic translation Initiation Factor 4E) complex, which is responsible for translation initiation. If a miRNA matches targets complimentary in its central region (9–11 nucleotides), mRNA can be cleaved by the endonuclease activity of AGO2 [60,65,66,67,68]. The canonical pathway of microRNA biogenesis is illustrated in Figure 1.

## 4. microRNA in AML

AML is a malignant clonal disorder of hematopoietic stem cells [69]. It is characterized by the uncontrolled clonal proliferation of abnormal progenitor cells in the bone marrow and blood [70]. Specific DNA alterations that affect the functions of normal bone marrow cells can cause them to transform into leukemia cells [71]. The majority of patients diagnosed with AML undergo a treatment that involves chemotherapy or undergo hematopoietic stem cell transplantation (HSCT), and certain subtypes of AML are treated with targeted therapies [72]. However, despite the advancements made in the past few years, the overall five-year survival rate continues to be disappointing [73,74]. According to the National Cancer Institute, the 5-year relative survival rate for AML was 31.9% during 2014–2020 [75]. Research has demonstrated that non-coding RNAs are intricately linked to the development and progression of AML, highlighting their significant role in the disease’s pathogenesis [76,77].

Altered expression levels of miRNAs are recognized as a significant mechanism in the pathogenesis of AML [78]. A precise understanding of these small molecules’ role is important for diagnosing, prognosticating, and developing therapies for AML patients [79]. This study reviews the role of miRNAs as potential molecular targets in AML. The articles for this review were selected based on the following inclusion criteria: (1) studies focused on AML; (2) examinations of miRNA expression levels using techniques such as reverse transcription quantitative polymerase chain reaction (RT-qPCR), sequencing, microarray technology, or data obtained from relevant databases; (3) the relationship between miRNAs and their target genes analysed using dual luciferase assays or databases (e.g., TargetScan, miRDB, ENCORI); (4) studies that included both a study and a control group; and (5) articles and abstracts published within the last ten years, from which data on miRNA alterations were collected [80,81,82]. The data presented in Table 2 and Table 3 refer to articles describing the expression of miRNA and their target genes. Table 2 focuses on downregulated miRNAs in AML, and Table 3 refers to upregulated miRNAs in AML. In order to simplify the text, miRNA names were shortened, and the “hsa-” prefix referring to Homo sapiens was removed. The growth of molecular biology has been striking, and the number of articles on miRNA has significantly increased. The selection of relevant articles was facilitated using the PICO tool https://jefflibraries.libguides.com/PICO/WhyPICO (accessed on 28 March 2025).

Dysregulated miRNA expression can result from various genomic changes in AML, including deletions, insertions, translocations, and, rarely, somatic copy number alterations of miRNA genes [16,83]. Deletions or copy loss of miRNA genes can lead to decreased miRNA expression, while amplifications or copy gains of miRNA genes can result in increased miRNA expression, which is recognized to be involved in AML development. For example, 5q deletion, a frequent alteration in AML and MDS, has been correlated with significantly downregulated miR-146a expression [84]. Common alterations in AML, such as loss of 7q or *CEBPA* impairment, have been associated with suppressed miR-29b [85]. Additionally, CEBPA loss has been linked to reduced miR-34a expression, a tumour suppressor in AML [86]. Translocations may lead to dysregulated expression of miRNA genes located near the breakpoints. For instance, elevated miR-125b expression has been observed with coexisting translocation t(2;11)(p21;q23) [87]. Furthermore, oncoproteins arising from fusion genes, which are common chromosomal alterations in AML, can lead to changes in miRNA expression, as observed in cases of *MLL*-rearranged AML, with upregulated miR-17-92 cluster and miR-9 [88,89].

Other important mechanisms that alter miRNA expression include epigenetic modifications, such as DNA methylation. For instance, improper *EVI1* expression impedes myeloid cell differentiation, partly through DNA hypermethylation and downregulation of miR-9 [90]. The expression levels of miR-126/126* might be partially influenced by the process of methylation in AML [91]. Also, methylation deregulation of miR-193a is associated with myeloid leukemogenesis [92].

It is also suggested that altered miRNA expression may be linked to transcription factors. For example, E2F1 is proposed as the miR-223 gene’s transcriptional inhibitor in AML [93]. Moreover, changes in chromatin accessibility, whether global or specific, can influence miRNA expression [94].

While mutations in the sequences of mature miRNAs are unlikely to directly alter their expression levels, such mutations could impact the specificity of target binding. For example, in the case of miR-142-3p, single-nucleotide variants (SNVs) that may affect target binding have been reported, although such occurrences are rare. On the other hand, polymorphisms in the 3′ UTR of miRNAs occur more frequently and can contribute to AML by regulating multiple genes [16]. Furthermore, the relationship between AML cell metabolism and miRNA expression could have an important impact on the advancement of the disease [95].

**Figure 2:**
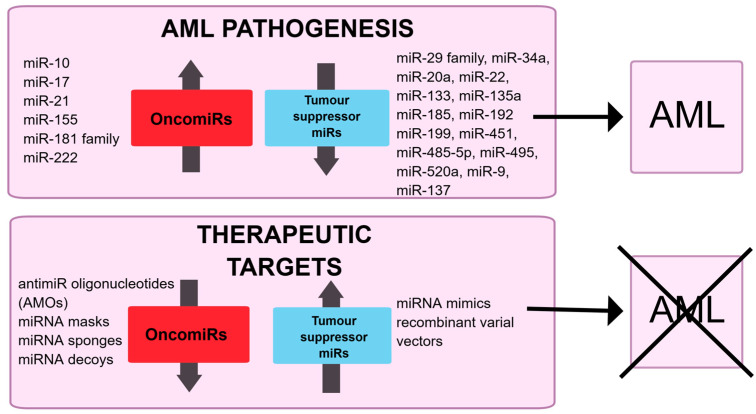
Schematic illustration of the potential role of miRNA in AML pathogenesis and their possible modulation in therapeutics.

Understanding the role of miRNA in AML is crucial for selecting suitable miRNA candidates as potential therapeutic targets. When microRNAs that function as oncomiRs are overexpressed, they can contribute to the development of AML. Therefore, as a therapeutic approach, these miRNAs should be downregulated to prevent disease progression. Conversely, miRNAs that act as tumour suppressors, when downregulated, can also promote disease development. In this case, these miRNAs should be overexpressed to help prevent AML [96]. There are some possible strategies which can be used to modulate miRNA expression, as shown in Figure 2. The promise of miRNA-based therapies is gaining more interest in the fight against various cancers. As of now, specific RNA-based therapies have received approval for medical use, but none have been approved for AML yet. So far, such treatments have neither undergone phase III clinical trials nor been approved by the U.S. FDA for clinical use [97].

Many studies describe the expression of miRNA in AML. Some of them are well studied, and their function in AML is understood. Nevertheless, studies on miRNAs are ongoing, and new reports continue to arise in the meantime, which is why little is known about certain miRNAs in AML, and the role of some miRNAs is still being investigated. Sometimes, their function is confusing, so collecting data on miRNA is crucial for considering their role. miRNAs described below are characterized by altered expression in AML. This review presents their role mostly on AML cells and describes their function in that disease.

Numerous papers have shown that the miR-29 family is significantly downregulated in AML, suggesting its role as a tumour suppressor in this disease. Wang et al. correlated negatively miR-29a relative expression with lncRNA XIST and *MYC* expression. They demonstrated that knocking down XIST led to an increased expression of miR-29a and a decreased expression of both MYC protein and mRNA, as well as reduced the tumorigenic ability of AML cells (KG-1) in vivo in mice [98]. Similarly, Tang et al. reported a negative correlation between expression levels of miR-29b-3p and both the mRNA and protein levels of HuR. The overexpression of miR-29b-3p led to a reduction in cell migration and cell proliferation, a decrease in colony-forming ability, and an increased apoptosis, along with K56a and U937 cell cycle arrest [99]. Furthermore, Randazzo et al. observed the co-occurrence of overexpressed miR-29a, miR-29c, and *DNMT3A* mutations in AML in contrast to patients without *DNMT3A* mutations [100]. Ngankeu et al. identified a germline polymorphism (a T base deletion) in the miR-29b-1/miR-29a cluster, which occurs more frequently in patients with core-binding factor AML (CBF-AML) [101].

miR-34a is generally considered a tumour suppressor in AML. Research has provided that the expression of miR-34a in AML is downregulated. Higher expression levels of miR-34a were present in AML patients with a platelet count lower than 34.5 × 10^9^/L and in those who achieved earlier complete remission (CR), as showed Abdellateif et al. Conversely, lower expression levels of miR-34 were observed in AML patients who were refractory to treatment. No correlation was found between the expression level of miR-34a and overall survival (OS) or event-free survival (EFS) [102]. An inverse correlation has been noted between the expression of miR-34a and PD-L1 in AML. Upregulation of miR-34a led to reduced cell surface PD-L1 expression, inhibited PD-L1 surface expression induced by IFN-γ in AML cells (HL-60), inhibited apoptosis of PD-L1+/CD8+ T cells, and disrupted IL-10 production in AML cells treated with IFN-γ [103]. Furthermore, the expression level of miR-34a was found to be lower in AML patients with intermediate and poor prognoses compared to healthy controls and AML patients with good prognoses. Overexpressed miR-34a led to suppressed KG-1a cells, inhibited *DHAC2* expression and triggered the death of leukemic stem cells (LSCs). In vivo, upregulation of miR-34a was shown to prolong survival and inhibit weight loss in mice transfected with LSCs [104]. Higher expression of miR-34a has been correlated with a favourable cytogenetic risk, while lower expression was associated with the M5 AML subtype [105]. Wen et al. provided evidence for the pivotal function of LSCs eradication of miR-34c-5p. Mice treated with engineered exosomes containing miR-34c-5p demonstrated prolonged survival, accompanied by a decrease in tumor burden, compared to other treated groups, indicating that these engineered exosomes might potentially serve an inhibitory function in the AML progression [106]. While miR-34c is also noted as a downregulated miRNA in AML, its suppressive function is not as clear-cut as that of miR-34a. Peng et al. associated lower miR-34c-5p expression levels with an adverse prognosis in AML and negatively correlated expression of miR-34c-5p with RAB27B mRNA and protein expression levels [107]. Similarly, Yang et al. showed that lower miR-34c expression levels were associated with significantly shorter OS in AML patients, indicating that miR-34c has the potential to emerge as a valuable biomarker [108]. The restoration of miR-34a expression has been proposed as a potential therapeutic strategy to inhibit tumour growth in patients with solid tumours [109].

Several studies have shown that the expression of miR-142 is downregulated, suggesting its role as a tumour suppressor in AML. Zhang et al. negatively correlated miR-142-3p expression with the expression of both protein and mRNA of the target HMGB1. Additionally, the expression of miR-142-3p was lower in cell lines resistant to the drug (HL-60/ATRA and HL-60/ADR), compared to HL-60. Overexpression of miR-142-3p increased the sensitivity of HL-60/ATRA and HL-60/ADR cells, promoting apoptosis in these cells [110]. Elsewhere, miR-142-5p relative expression was found to be significantly reduced in THP-1, HL-60, TF-1, NB4, and U937 cell lines; it was also demonstrated that miR-142-5p is inversely correlated with *PFKP* (platelet isoform of phosphofructokinase) expression, and with lncRNA XIST expression, compared to the control cell line (HS-5). In their study, lncRNA knocking down increased the expression of miR-142-5p, which resulted in the downregulation of *PFKP* in AML cells (U937 and THP-1) and inhibited cell cycle, proliferation, viability and colony formation. This also induced apoptosis in AML cells. Conversely, knocking down miR-142-5p led to AML progression, highlighting the role of the lncRNA XIST/miR-142-5p/PFKP axis in this disease [111]. miR-142 was also found to be downregulated in pediatric samples compared to healthy samples, and its expression was negatively correlated with the expression of circ-004136 [112]. Moreover, miR-142 expression has been described as downregulated in chronic myeloid leukemia (CML), miR-142 knockdown in mice has been linked to the development of a blast crisis [113].

Research indicates that miR-9 expression is reduced in AML, suggesting its function as a tumour suppressor. Liu et al. showed that overexpression of miR-9 enhances daunorubicin sensitivity in AML cells. Additionally, Zhu et al. found a negative correlation between miR-9 expression and *CXCR4* expression. miR-9 has been shown to reduce the proliferation and mobility of AML cells while increasing their apoptotic rate [114,115,116].

Recent studies also suggest a suppressive role for miR-20 in AML. Studies show that the relative expression of miR-20a-5p is decreased in AML. Bao et al. reported that lower relative expression of miR-20a-5p, according to the median value, is associated with shorter OS in AML patients compared to higher expression levels. They also found an inverse correlation between expression levels of miR-20a-5p and protein phosphatase 6 catalytic subunit, *PPP6C*. Upregulation of miR-20a-5p led to the inhibition of proliferation in AML cells (THP-1, U937), induction of cell cycle arrest, and increased apoptosis. An in vivo study showed that miR-20a-5p upregulation in mice resulted in smaller tumour sizes [117]. Ping et al. reported that the upregulation of miR-20a-5p inhibits the growth of AML cells through circRNA circ_0009910 [118]. In addition, miR-20a, along with miR-106b and miR-125b, contributes to the degradation of the PML-RARA oncoprotein [119].

A recently published article found that miR-103a-2-5p has tumor suppressive properties. The relative expression level of miR-103a-2-5p was found to be lower in samples from AML patients and AML cell lines (OCI-AML3, MOLM-13, THP-1, OCI-AML2 and MV4-11) in contrast to samples from healthy donors and normal cells (HS-5). Additionally, there was a negative correlation between the level of miR-103a-2-5p and the expression of its target, LILRB3, at both the mRNA and protein levels. Higher expression levels of miR-103a-2-5p were associated with longer OS. miR-103a-2-5p overexpressed inhibited proliferation, promoted cell cycle arrest, significantly increased the apoptosis rate and reduced migration of AML cells, and attenuated their clonality in AML cells (THP-1, MV4-11). An in vivo study revealed slower tumour growth in mice injected with miR-103a-2-5p encapsulated in cationic liposomes (CLPs) [120].

Some studies suggest that miR-133 and miR-135a may play a suppressive role in AML. Research indicates that miR-133 and miR-135a expression levels are downregulated [121,122,123,124]. Cheng et al. negatively correlated the expression of these miRNAs with their target gene, *CDX2*. In HL-60 AML cells treated with all-trans-retinoic acid (ATRA), there was a significant induction in the expression of both miRNAs. Overexpression of these miRNAs led to reduced proliferation of AML cells (NB4, HL-60). Among various AML subtypes, in M1, M2, and M3, the expression levels of both miRNAs were observed to be higher compared to other subtypes. Furthermore, the study showed that patients who achieved complete remission (CR) exhibited higher relative expression of these miRNAs compared to those who did not, correlating with a better leukemia-free survival rate [125]. According to databases by Wang et al., the target genes for miR-133 include *ZC3H15* and *BCLAF1*. An in vitro study using NB4 cells revealed that introducing miR-133 reduced the capacity for proliferation and accelerated apoptosis [123]. In addition, patients with a favourable karyotype demonstrated elevated expression of miR-133, which correlated with longer OS and relapse-free survival (RFS) [121]. A negative correlation was also observed between expression levels of miR-135a and *HOXA10*. In their examination of AML blood samples and AML cell lines (HL-60, AML193, AML2, and AML5), they noted decreased relative expression of miR-135a compared to normal blood samples and the normal cell line HS-5. Higher expression levels of miR-135a were associated with a better prognosis, that is, with longer OS in AML patients. Overexpression of miR-135a significantly elevated cellular apoptosis and suppressed the proliferation and cell cycle of HL-60 and AML5 cells [124]. Liu et al. also showed that miR-133 targets the *CXCL12* gene, and inhibiting miR-133 expression can enhance the sensitivity of HL-60/ADR cells to daunorubicin [126].

miR-137 is reported as downregulated and suggested to act as a tumour suppressor in AML. miR-137 expression was found to be negatively correlated with *c-KIT * expression, and restoring miR-137 expression inhibited proliferation and promoted differentiation [127]. Additionally, Wang et al. inversely correlated the expression levels of miR-137 and *TRIM25*. Overexpressing miR-137 resulted in reduced proliferation, migration, and invasion of AML cells [128].

Similarly, miR-148 is also described as downregulated and acts as a tumour suppressor in AML [129,130]. Wang et al. reported that the expression of its direct target, *DNMT1*, was inversely correlated with miR-148 expression. Moreover, overexpression of miR-148 inhibited the proliferation and increased the apoptosis of AML cells [130].

Research indicates that miR-185 is downregulated in AML. Pang et al. highlighted the role of miR-185 as a tumour suppressor, reporting significantly lower levels of relative miR-185 expression in specimens collected from AML patients and AML cell lines compared to specimens from healthy individuals and normal cells. They found that *GPX1* expression was negatively correlated with miR-185-5p expression. Overexpression of miR-185-5p decreased the viability, proliferation, invasion, and apoptosis of AML cells (HL-60, KG-1) [131]. Similarly, Zhang et al. also described downregulated miR-185 expression in AML samples and cell lines compared to normal samples, noting its regulation by TUG1 lncRNA [132].

The expression of miR-192 is found to be downregulated in AML. A study conducted by Wu et al. [133] examined the role of the circ_0001602/miR-192-5p/ZBTB20 axis, revealing that circ_0001602 and *ZBTB20* expression levels were upregulated, while miR-192-5p expression levels were downregulated in AML cell lines (THP-1, HL-60, NB4) and patient samples compared to samples from healthy individuals and the HS-5 cell line. Knockdown of circ_0001602 resulted in reduced proliferation of AML cells and induced apoptosis and cell cycle arrest. However, a deficiency of miR-192-5p abolished this effect. Moreover, overexpression of miR-192-5p reduced cell viability, inhibited cell cycle progression, and induced cell apoptosis in AML cell lines; however, this effect was diminished after the addition of *ZBTB20*. This evidence suggests a sponge effect of circ_0001602 affecting this miRNA; the study also highlights the downstream influence of miRNA on its target gene, which overall indicates a pivotal role of the examined axis in the progression of AML. Additionally, the expression of miR-192 was downregulated in AML pediatric patients compared to healthy controls [134]. Ke et al. reported downregulated expression of miR-192 in AML patients compared to controls and its binding to *CCNT2*, indicating that miR-192 functions as a tumour suppressor. Overexpression of miR-192 induced cell cycle arrest, apoptosis, and cell differentiation [135].

Research shows that miR-451 is downregulated in AML and suggests its role as a tumour suppressor. Krakowsky et al. found an inverse correlation between the expression of miR-451 and *MDR1* expression, suggesting that miR-451 has the potential to enhance the chemotherapy sensitivity of *FLT3*-ITD+ cells by targeting *MDR1*. Li et al. found that miR-451a was one of the significantly decreased miRNAs, alongside miR-185-5p, miR-443b-3p, miR-199a-5p, and miR-151a-3p among the examined miRNAs. Furthermore, Song et al. suggested the role of the hnRNP A1/miR-451/c-Myc axis in AML development. Su et al. discovered that miR-451 targets *YWHAZ* and suppresses YHWAZ-AKT signalling pathway in AML. Overexpression of miR-451 resulted in increased apoptosis rates of AML cells and inhibited their proliferation [136,137,138,139,140].

Recently, studies by Wu et al. and by Xie et al. have shown that miR-455-3p is downregulated in AML. The first study indicates that miR-455-3p functions as a tumour suppressor in AML [141,142]. The second study demonstrated a negative correlation between miR-455-3p and its target gene, *UBN2*. AML cells transfected with the miR-455-3p mimic exhibited decreased proliferation, inhibited cell viability, induced cell cycle arrest, and induced both apoptosis and autophagy. The study highlighted the critical regulatory role of miR-455-3p in AML through the mediation of peroxisome proliferator-activated receptor α (PPARα) [142].

miR-485-5p was identified as downregulated and suggested to function as a tumour suppressor in AML. Wang et al. found a negative correlation between miR-485-5p expression and its target gene, *SALL4*. The upregulation of miR-485-5p elevated the apoptotic rate and attenuated the proliferative ability of AML cells (AML5 and U937) [143,144].

Similarly, miR-495 was described as downregulated, and Wang et al. confirmed its suppressive role in AML [145,146,147,148]. Lei et al. reported that the relative expression levels of miR-495-3p and miR-543 were lower in AML bone marrow samples and AML cells (HL-60 and Kasumi-1) compared to control bone marrow samples and control cells (HS-5). They found that the expression of the target gene *PDK1* was negatively correlated with the levels of these miRNAs. In AML cell lines treated with matrine, the expression levels of miR-495-3p and miR-543 increased. Overexpression of these miRNAs led to attenuated viability, promoted apoptosis, induced cell cycle arrest, and inhibited glycolysis, indicating their regulatory role in AML under matrine treatment [146].

miR-520a is also downregulated in AML and is recognized as a tumour suppressor, according to studies by Chen et al. and Xiao et al. The first author correlated reversely the expressions of miR-520a-3p and *MUC1*, demonstrating that overexpression of miR-520a-3p inhibited proliferation and induced apoptosis of AML cells (THP-1) [149]. Xiao et al. similarly showed that the overexpression of miR-520a reduced proliferation and cell viability while inducing apoptosis. The functional study demonstrated that the anti-proliferative effects of miR-520a were primarily due to its ability to block the PI3K/AKT signalling pathway [150].

The expression of miR-30e-5p was also found to be downregulated and negatively correlated with *CYB561* expression. Overexpression of miR-30e-5p delayed the onset of leukemia in mice with *KMT2A::MLLT3*. Additionally, upregulation of miR-30e-5p also impaired the self-renewal abilities of LSCs. Notably, no significant correlation was reported in the case of miR-30e-3p in AML samples compared to healthy samples [151].

Ye Q et al. reported that the relative expression of miR-211-5p was downregulated in pediatric AML samples compared to control samples and was negatively correlated with the expression of its target gene, *JAK2*. Overexpression of miR-211-5p, induced by cryptanshinone (CPT), led to increased apoptosis of LSCs, decreased LSC proliferation and viability, and reduced concentrations of proinflammatory cytokines (IL-1β, TNF-ɑ). Inhibition of miR-211-5p resulted in the opposite effect, highlighting its regulatory role in CPT-treated AML cells [152].

Xie et al. showed that miR-409-3p was downregulated in the THP-1 cell line compared to the normal cell line (HS-5). miR-409-3p negatively regulated its target gene, *RAB10*. Overexpression of miR-409-3p reduced the proliferation of THP-1 cells and increased apoptosis, thus exerting its suppressive effect in AML [153].

Wang et al. demonstrated that the expression of miR-454-3p was significantly decreased in AML cells, and the target for miR-454-3p is *ZEB2*. Overexpression of miR-454-3p in THP-1 cells resulted in reduced cell viability, induced cell cycle arrest, and promoted apoptosis and autophagy, confirming its role as a tumour suppressor in AML [154].

The circPLXNB2/miR-654-3p/CCND1 axis was examined in AML by Wang et al. They found that the relative expression of miR-654-3p in AML patients was downregulated, and *CCND1* was identified as its direct target. Elevated expression of miR-654-3p led to decreased proliferation of HL-60 and Kasumi-1 cells, increased apoptosis, and induced cell cycle arrest [155].

miR-1294 was downregulated in AML and negatively regulated by circFN1, as described by Wang et al. AML cells (HL-60) transfected with the miR-1294 mimic exhibited significantly lower cell proliferation and invasion abilities compared to those transfected with the negative control (NC) mimic. Moreover, apoptosis was higher in miR-1294 mimic-transfected cells than in the NC mimic-transfected cells. The study confirmed that miR-1294 negatively regulates its target gene, *ARHGEF10L*, thereby demonstrating the regulatory role of the circFN1/miR-1294/ARHGEF10L axis in AML [156].

Conversely, miR-10 is reported to be upregulated in AML, and its role is characterized as oncogenic. Zhi et al. found a correlation between higher expression levels of miR-10a-5p and shorter OS. Additionally, Wang et al. reported overexpressed miR-10b in pediatric specimens in contrast to the ITP group and was negatively correlated with *HOXD10* expression. Overexpression of miR-10b accelerated the proliferation of AML cells (HL-60), increased the number of cells in the S phase, and decreased the apoptosis rate [157,158,159,160].

miR-21 is described as upregulated and acting as an oncogene in AML. Li et al. negatively correlated miR-21 expression with *KLF5* expression. miR-21 inhibition decreased AML cell proliferation. Moreover, overexpression of miR-21 resulted in decreased *KLF5* expression, thereby promoting tumorigenesis in vivo [161]. Higher levels of miR-21 expression were associated with shorter OS and RFS in AML patients [162]. Notably, in patients with mutated *NPM1*, the expression of miR-21 was markedly increased [163].

Similarly, miR-17 was also found to be upregulated in AML [164,165,166]. Studies indicate that miR-17-5p is upregulated in AML cells (THP-1 and HL-60) compared to normal cells (HS-5). The expression levels of its target gene, *JAK1*, and the lncRNA SUCLG2-AS1 are negatively correlated with miR-17-5p, as shown by Liu et al. Their research demonstrated that SUCLG2-AS1 modulates the expression of *JAK1* and influences the leukemic (THP-1, HL-60) cells’ apoptosis, proliferation, invasion, and migration via miR-17-5p. Furthermore, miR-17-5p modulates apoptosis, invasion, and migration of AML cells through *JAK1*, highlighting the regulatory role of the lncRNA SUCLG2-AS1/miR-17-5p/JAK1 axis in AML [165]. Wang et al. also reported an upregulation of miR-17-5p expression in AML samples compared to healthy volunteers, noting that higher expression of miR-17-5p correlated with shorter RFS and OS in AML patients. Additionally, the research indicates a negative correlation between miR-17-5p and *BECN1* expression. After treatment with vitamin D, miR-17-5p expression decreased and reduced AML cell (HL-60) proliferation. [166].

According to Huang et al., miR-93 is upregulated in AML and activates the PI3K/AKT pathway via *DAB2*. Knocking down miR-93 inhibited AML cell proliferation. Moreover, miR-93 downregulation led to AML cells’ apoptosis and cell cycle arrested. Conversely, overexpression of miR-93 led to an increase in tumour size in mice in vivo [167].

A recent study indicated that the relative expression of both miR-106b-5p and its target, Rab10 mRNA and protein, is upregulated in AML. In patients at the initial stage of AML, lower expression levels of miR-106b-5p and Rab10 protein were associated with higher survival rates [168].

miR-126 is upregulated in inv(16)(p12q22) AML, as demonstrated by Zhang et al. A decrease in miR-126 expression resulted in inhibited AML cell survival and activity in vivo [169].

miR-146 was also described as being upregulated in AML. Wang et al. found that the relative expression of miR-146a was significantly increased in pediatric AML patients compared to healthy children. Moreover, the miR-146a expression level was negatively correlated with the expression of its target, *CNTFR*. When miR-146a was downregulated, there was a significant decrease in proliferation, increased apoptosis, and reduced migration of leukemic cells (HL-60) [170]. Li et al. confirmed the upregulation of miR-146b-5p in examined patients with AML in contrast to healthy samples of RNA collected from extracellular vesicles (EVs) in serum. Additionally, their study identified other upregulated miRNAs, including miR-10a-5p, miR-155-5p, miR-100-5p, and let-7a-5p, while downregulated miRNAs included miR-185-5p, miR-4433b-3p, miR-199a-5p, miR-451a, and miR-151a-3p [171].

miR-155 is similarly recognized as upregulated in AML, suggesting an oncogenic role in AML [172,173,174,175,176]. Garavand et al. observed no correlation between miR-155-5p expression and either *KRAS* or *CREB* expression [172]. Xue et al. found that the relative expression of miR-155 was upregulated in samples from AML patients and cell lines compared to healthy volunteers and CML cell lines, with a negative correlation to relative *SHIP1* expression. After inhibition of miR-155, the proliferation of AML cells (THP-1, U937) decreased, and the apoptosis rate increased [173]. Hatem et al. also reported in AML an upregulation of miR-155 along with miR-10a and let-7a. [176].

miR-181 was identified as upregulated in AML and is thought to function as an oncomiR [177,178,179]. A study of the miR-181 family by Sue et al. found that the expression of miR-181a, miR-181b, miR-181c, and miR-181d was higher in AML samples compared to controls. This study indicates a negative correlation between these miRNAs and genes *PRKCD*, *CTDSPL*, and *CAMKK1*. The study highlighted the role of the miR-181 family in granulocytic and macrophage-like differentiation [179].

According to Gao et al., miR-1306-5p is increased in AML and acts as a tumour oncogene. The relative expression of *PHF6* was negatively correlated with miR-1306-5p expression levels. Downregulation of miR-1306-5p led to decreased proliferation and increased apoptosis of AML cells [180].

The function of miR-22 remains unclear. Many studies suggest that it acts as a tumour suppressor and report the downregulation of miR-22 in AML. On the other hand, a singular study indicates upregulation of miR-22 expression in AML. For instance, Jiang et al. found that the relative expression of miR-22 was significantly lower in de novo AML and negatively correlated with *CRTC1* and *FLT3* expression. Restoring miR-22 expression inhibited in vitro AML colony formation, promoted apoptosis of AML cells, and decreased AML cell viability and growth in the MONOMAC-6 cell line. In an in vivo study, it was reported that the upregulation of miR-22 significantly inhibited leukemogenesis in mice, supporting its anti-tumour role in AML [181]. Similarly, Shen et al. reported downregulated expression of miR-22 in de novo AML, finding a reverse association between expression of miR-22 and *MECOM(EVI1),* and a positive correlation with the expression level of transcription factor PU.1. The study proved that upregulation of miR-22 alleviated differentiation inhibition in AML bone marrow blasts, resulting in a notable reduction in the HL60 and THP1 cells growth [182]. Qu et al. also noted downregulated miR-22 expression in AML and associated it with worse OS and RFS in AML patients. Additionally, increased levels of miR-22 were observed in pre- and post-treated specimens and was linked with the achievement of CR [183]. On the other hand, Yao et al. presented upregulated expression of miR-22-3p and miR-22-5p in AML [184]. Although the expression of miR-22 is described differently in various research studies, the significant majority of them focus on the anti-tumour function of this miRNA.

The expression of miR-92a shows a dual role in studies on AML, appearing both upregulated and downregulated. For example, Gu et al. reported significantly lower relative levels of miR-92a expression in AML cell lines (HL-60 and THP-1) compared to a normal cell line (HS-5). They negatively correlated the expression of miR-92a with *MTHFD2* expression, suggesting a suppressive role for miR-92a in AML. Moreover, the upregulation of miR-92a inhibited AML cell proliferation and induced a surge in apoptosis by regulating *MTHFD2* [185]. On the other hand, Su et al. highlighted the role of the circ_0002232/miR-92a-3p/PTEN axis in AML, showing that in AML patients, miR-92a-3p was upregulated in contrast to healthy donors. Sue et al. correlated inversely the expressions of miR-92a and *PTEN*. [186]. Saadi et al. described increased expression of miR-92a and miR-181a in AML patients compared to the control group [187]. Additionally, Rashed et al. demonstrated that the post-induction level of miR-92a expression was significantly higher in patients who achieved CR compared to those who did not, indicating that miR-92a has the potential to be an effective biomarker [188].

miR-199 is predominantly recognized as a tumour suppressor in AML; however, there is some limited evidence suggesting that it may act as an onco-miR in a specific context. Most of the research indicates that the expression level of miR-199 is downregulated in AML. For example, Qi and Zhang found a correlation between decreased miR-199 expression and poor risk stratification in pediatric AML patients [189]. Likewise, Li et al. demonstrated that patients with relapsed/refractory AML had lower expression of miR-199a-5p than those who achieved CR. They also discovered that miR-199a-5p directly targets *DRAM1*, and overexpression of miR-199a-5p inhibited autophagy and reduced chemoresistance upon adriamycin treatment [190]. Ellson et al. associated miR-199 higher expressions with improved OS and EFS in AML patients [191]. Favreau et al. noted that lower expression of miR-199b correlated with shorter OS in AML patients and indicated its prognostic significance in the FAB-M5 subtype [192]. Conversely, Alemdehy et al. suggested that miR-199a-3p functions as an onco-miR in AML, showing that this miRNA caused AML in an in vivo mice model [193].

In contrast, the role of miR-222 is more frequently reported as oncogenic in AML studies; however, there is one article that indicates its potential suppressive function in AML. Yuan et al. reported decreased miR-222-3p expression in isolated exosomes secreted by bone marrow mesenchymal stem cells (BM-MSC) in AML patients compared to the exosomes from healthy donors. They found that upregulation of miR-222-3p elevated the Th1/Th2 ratio, confirmed in vivo, and induced apoptosis of AML cells (HL-60). The expression of miR-222-3p was also found to be negatively correlated with IRF2 expression [194]. Pei et al. reported an increase in the relative expression of both miR-221 and miR-222 in AML samples compared to healthy ones, noting a negative correlation between miRNA expression and *YOD1* expression. Downregulation of miR-221/222 increased the p53 protein level and reduced its ubiquitination [195]. Liu et al. observed higher relative expression of miR-222-3p in AML cell lines (NB4, U937, KG1a, THP1) compared to peripheral blood mononuclear cells (PBMCs) from healthy controls, identifying *AXIN2* as a target for miR-222-3p. Downregulation of miR-222-3p led to decreased viability and induced apoptosis of AML cells (NB4 and U937) [196]. Likewise, Pavlovic et al. also reported upregulation of miR-222 in AML, supporting its oncogenic function [197].

miR-361 has been reported as both downregulated and upregulated in AML. Xu et al. reported decreased relative expression of miR-361-3p in *KMT2A*-rearranged AML patients and AML cell lines (THP-1, HL-60, KG-1A, KO52) compared to control subjects and the cell line (HS-5). They confirmed a negative correlation between the expression of miR-361-3p and KMT2A. Overexpression of miR-361-3p decreased proliferation, migration, and invasion of AML cells (HL-60) [198]. Conversely, Liu et al. reported an increased relative expression of miR-361-3p in AML patients and the AML cell line (HL-60) compared to healthy donors, indicating its binding to *BTG2*. The downregulation of miR-361-3p expression reduced proliferation and increased cell apoptosis under 9s-HODE, a major active derivative of linoleic acid, suggesting an oncogenic role of miR-361-3p in AML [199].

**Table 2:** List of downregulated miRNAs in AML and their target genes; effect on AML cells while microRNA is overexpressed; type and size of microRNA expression study/control group.

↓-miRNA	Target	Effect on AML/Leukemic Cells When miRNA Is Overexpressed	Method (Study Group/Control Group or Cell Lines)	Reference
miR-9	*CXCR4*	Reduction of proliferation and mobility of AML cells. Increased apoptosis rate of AML cells.	RT-qPCR (36 AML BM/10 BM; NB4, HL-60, Kasumi-1, SKNO-1, KG-1a/normal CD34+ cells), miRNA mimics (Kasumi-1, SKNO-1)	[116]
miR-20a-5p	*PPP6C*	Inhibition of cell proliferation, induction of cell cycle arrest and apoptosis. Decrease in tumor size in vivo.	RT-qPCR (61 AML BM/61 BM; Kasumi-1, THP-1, U937, HL-60/HS-5), miRNA mimics (THP-1, U937)	[117]
miR-22	*CRTC1* *FLT3*	Inhibition of cell colony forming, viability, and growth. Inhibition of leukemogenesis in mice in vivo. Promotion of cell apoptosis.	RT-qPCR (42 AML MNC/5 MNC), miRNA mimics (MONOMAC-6, THP-1, KOCL-48)	[181]
	*EVI1*	Relieved blockage in the differentiation of bone marrow blasts. Inhibition of cell growth.	RT-qPCR (79 AML PB MNC/114 PB MNC; 41 AML BM MNC/8 BM MNC; 50 AML BM CD34+/10 BM CD34+), miRNA mimics (HL-60, THP1)	[182]
miR-29a	*MYC*	Inhibition of cell tumorigenic ability in mice in vivo.	RT-qPCR (62 AML BM MNC/20 BM MNC; KG-1/BM MNC), miRNA mimics (KG-1)	[98]
miR-29b-3p	*HuR*	Inhibition of cell proliferation, colony-forming capability, and cell migration. Promotion of cell cycle arrest at G0/G1 phase and apoptosis.	RT-qPCR (K562, NB4, U937, K562/G01, Kasumi-1, HL60/PB MNC), miRNA mimics (K562, U937)	[99]
miR-30e-5p	*CYB561*	Impaired cell self-renewal. Inhibited onset of *KMT2A::MLLT3*-driven leukemia in mice in vivo.	RT-qPCR (29 AML BM MNC/6 BM MNC), miRNA mimics (*KMT2A::MLLT3* AML cells)	[151]
miR-34a	*PD-L1*	Reduction in cell surface PD-L1 expression. Reduction in INF-γ-induced PD-L1 surface expression, apoptosis of PD-L1/CD 8+ T cells and IL-10 production upon IFN-γ	RT-qPCR (13 AML BM/5 BM), miRNA mimics (HL-60, Kasumi-1)	[103]
	*DHAC2*	Suppressed proliferation. Induced LSC death. Prolonged survival in AML mice in vivo.	RT-qPCR (30 AML BM/10 BM), miRNA mimics (KG-1a)	[104]
miR-92a	*MTHFD2*	Inhibition of cell proliferation and promotion of apoptosis.	RT-qPCR (HL-60, THP-1/HS-5), miRNA mimics (HL-60, THP-1)	[185]
miR-103a-2-5p	*LILRB3*	Inhibition of cell proliferation, migration, and clonality. Promotion of apoptosis and cell cycle arrest.	RT-qPCR (30 AML BM MNC/BM MNC), miRNA mimics (THP-1, OCI-AML2, OCI-AML3, MV4-11)	[120]
miR-133	*ZC3H15* *BCLAF1*	Inhibition of AML cell proliferation. Acceleration of AML cell apoptosis.	miRNA sequencing (102 AML samples/CD34+) *, miRNA mimics (NB4)	[123]
miR-135a	*HOXA10*	Inhibition of AML cell proliferation and cell cycle. Acceleration of AML cell apoptosis.	RT-qPCR (29 AML PB/11 PB; HL-60, AML193, AML2, AML5/HS-5), miRNA mimics (HL-60, AML5)	[124]
miR-133a miR-135a	*CDX2*	Inhibition of AML cell proliferation.	RT-qPCR (59 AML BM/9 BM), miRNA mimics (HEK-293, NB4, HL-60)	[125]
miR-137	*C-kit*	Inhibition of proliferation and promotion differentiation of AML cells.	RT-qPCR (49 AML BM/57 BM), miRNA mimics (Kasumi-1, K562)	[127]
	*TRIM25*	Inhibited AML cells proliferation, migration and invasion.	RT-qPCR (45 AML BM/45 BM; HEL, Kasumi-1, HL-60, MEG01/HS-5), miRNA mimics (Ksaumi-1, HL-60)	[128]
miR-142-3p	*HMGB1*	Improvement of drug sensitivity in AML cells.	RT-qPCR (23 AML PB MNC/15 PB MNC), miRNA mimics (HL-60/ATRA, HL-60/ADR)	[110]
miR-142-5p	*PFKP*	Inhibition of AML cell proliferation, viability, cloning, and cycle. Induction of AML cell apoptosis.	RT-qPCR (THP-1, HL-60, TF-1, NB4, U937/HS-5), miRNA mimics (THP-1, U937)	[111]
miR-148	*DNMT1*	Inhibition of proliferation and induction of apoptosis of AML cells.	RT-qPCR (80 AML BM MNC/20 BM MNC; U937, THP-1, Kasumi-1/20 BM MNC), miRNA mimics (U937, Kasumi-1)	[130]
miR-185-5p	*GPX1*	Inhibition of viability, proliferation, invasion, and promotion of apoptosis.	RT-qPCR (37 AML BM/37 BM; MOLM-14, HL-60, KG-1/HS-5), miRNA mimics (KG-1, HL-60)	[131]
miR-192-5p	*ZBTB20*	Inhibition of cell viability. Induction of cell apoptosis and cell cycle arrest.	RT-qPCR (52 AML BM/34 BM THP-1, HL-60, NB4/HS-5), miRNA mimics (THP-1, HL-60)	[134]
	*CCNT2*	Induction of cell cycle arrest, apoptosis, and cell differentiation.	RT-qPCR (10 AML BM/10 BM; NB4, HL-60/10 BM), miRNA mimics (NB4, HL-60)	[135]
miR-199a-5p	*DRAM1*	Reduction of chemoresistance upon ADM treatment. Inhibition of autophagy.	RT-qPCR (32 AML relapsed/refractor BM/11 complete remission BM), miRNA mimics (K562/ADM, K562)	[190]
miR-211-5p	*JAK2*	Reduction of AML cell proliferation, viability, and inflammation. Induction of AML cell apoptosis.	RT-qPCR (50 AML PB/50 PB), miRNA mimics (CPT treated LSCs)	[152]
miR-222-3p	*IRF2*	Increased Th1/Th2 ratio. Induction of cell apoptosis.	RT-qPCR (20 BM of AML patients and healthy donors), miRNA mimics (HL-60)	[194]
miR-361-3p	*KMT2A*	Reduction of cell proliferation, migration, and invasion.	RT-qPCR (30 AML PB/30 PB; HL-60, KG-1a, KO52, THP-1/HS-5), miRNA mimics (HL-60, H562)	[198]
miR-409-3p	*RAB10*	Inhibition of cell proliferation and induced apoptosis.	RT-qPCR (THP-1, NB4/HS-5), miRNA mimics (THP-1)	[153]
miR-451	*YWHAZ*	Suppression of AML cell proliferation. Increased AML cell apoptosis.	RT-qPCR (69 AML PB MNC/80 PB MNC; 56 AML BM MNC/9 BM MNC; 32 AML BM CD34+/9 BM CD34+), miRNA mimics (NB4, HL-60)	[140]
miR-454-3p	*ZEB2*	Inhibited viability and induced cell cycle arrest. Induction of apoptosis and autophagy.	RT-qPCR (NB4, THP-1, KG-1a, U937/PB MNC), miRNA mimics (THP-1)	[154]
miR-455-3p	*UBN2*	Inhibition of cell proliferation and viability. Induction of cell apoptosis, autophagy, and cell cycle arrest.	RT-qPCR (16 AML PB/16 PB; HL-60, Kasumi-1, KG1, THP-1, MV4-11/PB MNC), miRNA mimics (HL-60)	[142]
miR-485-5p	*SALL4*	Inhibition of cell proliferation and induction of cell apoptosis.	RT-qPCR (35 AML BM/35 BM; AML2, AML193, Kasumi-1, HL-60, AML5, U937/HS-5), miRNA mimics (AML5, U937)	[144]
miR-495-3p miR-543	*PDK1*	Inhibition of cell proliferation, cell glycolysis, viability under matrine. Induction of cell apoptosis and cell cycle arrest under matrine.	RT-qPCR (31 AML BM/31 BM; HL-60, Kasumi-1/HS-5), miRNA mimics (HL-60, Kasumi-1)	[147]
miR-520a-3p	*MUC1*	Inhibition of cell proliferation and induction of apoptosis.	RT-qPCR (25 AML PB/25 PB), miRNA mimics (THP-1)	[150]
miR-654-3p	*CCND1*	Inhibition of cell proliferation, induction of apoptosis and cell cycle arrest.	RT-qPCR (51 AML PB/51 PB; HL-60, Kasumi-1/HS-5), miRNA mimics (HL-60, Kasumi-1)	[155]
miR-1294	*ARHGEF10L*	Inhibition of AML cell proliferation and invasion. Induction of AML cell apoptosis.	RT-qPCR (16 plasma/16 plasma; HL-60/HS-5), miRNA mimics (HL-60)	[156]

*—data from GEO database; BM, bone marrow; MNC, mononuclear cells; PB, peripheral blood.

**Table 3:** List of upregulated miRNAs in AML and their target genes; effect on AML cells while microRNA is knockdown; type and size of microRNA expression study/control group.

↑-miRNA	Target	Effect on AML Cells While miRNA Is Downregulated	Method (Study Group/Control Group or Cell Lines)	Reference
miR-10b	*HOXD10*	ND	RT-qPCR (108 AML serum samples/25 serum samples)	[158]
miR-17-5p	*JAK1*	Inhibition of cell proliferation, migration, invasion, and promotion of apoptosis when lncRNA SUCLG2-AS1 overexpressed.	RT-qPCR (THP1, HL60/HS-5), miRNA mimics (THP-1, HL-60)	[165]
	*BECN1*	Inhibition of AML cell proliferation after treatment of vitamin D.	RT-qPCR (144 AML PB/45 PB), miRNA mimics (HL-60)	[166]
miR-21	*KLF5*	Reduction of AML cells proliferation.	RT-qPCR (SKM-1, HL-60/HS-5), miRNA mimics (SKM-1, HL-60)	[161]
miR-92a-3p	*PTEN*	ND	RT-qPCR (115 AML BM MNC/48 BM MNC)	[186]
miR-93	*DAB2*	Inhibition of AML cells proliferation. Promotion of cell cycle arrest and apoptosis of AML cells.	RT-qPCR (28 AML BM MNC/30 BM MNC), miRNA mimics (THP-1, HL-60, HS-5)	[167]
miR-106-5p	*RAB10*	ND	RT-qPCR (85 AML BM MNC/15 BM MNC)	[168]
miR-146a	*CNTFR*	Reduction of leukemic cells proliferation and migration. Increasing leukemic cells apoptosis.	RT-qPCR (11 AML BM/10 BM), miRNA mimics (HL-60)	[170]
miR-155	*SHIP1*	Inhibition of AML cell proliferation and promotion of apoptosis.	RT-qPCR (30 AML BM or PB MNC/10 BM MNC), miRNA mimics (U937, THP-1)	[173]
miR-181 family	*PRKCD* *CTDSPL* *CAMKK1*	Modulation of granulocytic and macrophage-like differentiation.	RT-qPCR (95 AML PB MNC/75 PB MNC; 36 AML BM CD34+/9 BM CD34+), miRNA mimics (HL-60)	[179]
miR-221 miR-222	*YOD1*	Increasing p53 protein level and reducing its ubiquitination.	RT-qPCR (18 AML PB/20 PB and 6 BM), miRNA mimics (U2OS, HCT116)	[195]
miR-222-3p	*AXIN2*	Decreased cell viability and induced apoptosis.	RT-qPCR (KG1a, NB4, U937, THP1/PB MNC), miRNA mimics (NB4, U937)	[196]
miR-361-3p	*BTG2*	Inhibition of cell proliferation and induction of apoptosis.	RT-qPCR (34 AML PB/5 PB; HL-60/5 PB), miRNA mimics (HL-60)	[199]
miR-1306-5p	*PHF6*	Inhibition of AML cell proliferation and induction of apoptosis rate.	RT-qPCR (48 AML BM/30 BM; HL-60, Kgla, K562, THP-1/HS-5), miRNA mimics (HL-60, K562)	[180]

BM, bone marrow; MNC, mononuclear cells; PB, peripheral blood; ND, no data.

### miRNA Associated with Genetic Abnormalities

According to the 2022 ELN recommendations, AML is classified into numerous subtypes with respect to genetic abnormalities, including: AML with recurrent genetic abnormalities, AML with mutated *TP53*, AML with myelodysplasia-related cytogenetic abnormality, and AML not otherwise specified (AML NOS). AML with recurrent genetic abnormalities constitute a large group of AML subtypes, which comprises: APL with t(15;17)(q24.1;q21.2)/*PML::RARA*, AML with t(8;21)(q22;q22.1)/*RUNX1::RUNX1T1*, AML with inv(16)(p13.1q22) or t(16;16)(p13.1;q22)/*CBFB::MYH11*, AML with t(9;11)(p21.3;q23.3)/*MLLT3::KMT2A*, AML with t(6;9)(p22.3;q34.1)/*DEK::NUP214*, AML with inv(3)(q21.3q26.2) or t(3;3)(q21.3;q26.2)/*GATA2*, *MECOM*(*EVI1*), AML with other rare recurring translocations, AML with mutated *NPM1*, AML with in-frame bZIP mutated *CEBPA,* and AML with t(9;22)(q34.1;q11.2)/BCR::ABL1. Some studies indicate the significance of miRNAs in the development of leukemia, particularly regarding distinct subtypes of the disease, which may prove valuable clinically in the future. Data is collected in Table 4.

A few reports indicate particular miRNAs which may be relevant in the t(8;21) AML subtype; for instance, miRNA let-7b, when overexpressed, is reported to reduce AML1-ETO protein expression. Additionally, miRNA let-7b is noted to inhibit the proliferation of t(8;21) AML cell lines and induce differentiation, indicating that this miRNA may play a role in the leukemic characteristics associated with the t(8;21)(q22;q22.1) chromosomal translocation [200]. miR-223 is reported as downregulated in t(8;21) AML and is identified as a direct target of the AML1/ETO oncoprotein. AML1/ETO alters the expression level of miR-223, ultimately restoring cell differentiation in cases of AML with t(8;21)(q22;q22.1) [201]. miR-9-1 is showed as downregulated in AML associated with the t(8;21) chromosomal translocation. *RUNX1::RUNX1T1* is regulated by miR-9-1, and silencing miR-9-1 has been shown to increase the oncogenic activity of *RUNX1::RUNX1T1*. Additionally, the miR-9-1 overexpression reduces proliferation and promotes differentiation of t(8;21) cell lines [202]. Higher expression levels of miR-126 are reported to be associated with poor prognosis in patients with t(8;21) AML [203]. miR-130a expression is presented as increased in t(8;21) AML, which induce apoptosis and differentiation of t(8;21) AML cells. The level of miR-130a is suggested to be important for t(8;21) AML maintenance [204]. miR-383 is presented as upregulated in AML1-ETO positive AML, and AML1-ETO regulates its expression. In turn, miR-383 negatively regulates THAP10 expression, which functionally inhibits leukemogenesis of t(8;21) AML [205].

In APL with fusion gene *PML::RARA*, miR-15b is described to have an essential function in the proliferation and differentiation of acute promyelocytic leukemia. Also, miR-15b is indicated as a tumour suppressor in APL, and its target gene is CCNE1 [206]. miR-125b is described in APL, and its overexpression in t(15;17) APL expands PML-RARA-induced leukemogenesis in vivo [207]. micoRNA-382-5p is important in the differentiation of ATRA-induced APL, which targets PTEN [208].

In AML with fusion gene *CBFB::MYH11*, miR-126 was examined in vivo. The study indicated that deleting miR-126 prevented the development of leukemia in 50% of *CBFB::MYH11* knock-in mice and significantly extended their survival. The study also revealed that abnormal expression of miR-126 significantly contributes to the onset of leukemogenesis and maintains AML induced by *CBFB::MYH11* [169].

In the context of AML with fusion gene *MLLT3::KMT2A,* miR-30e is described to play a crucial role in developing this AML subtype. In vivo, the study showed that the overexpression of miR-30e delays the development of *KMT2A::MLLT3*-driven leukemia [151].

AML with mutated *NPM1* showed upregulated miR-10a compared to *NPM1* wild-type AML, indicating its potential role in *NPM1*-mutated AML by targeting *MDM4* [209]. Moreover, miR-21 is reported to be overexpressed in *NPM1*-mutated AML, where it targets *PDCD4*. This abnormal expression is thought to contribute to the pathogenesis of *NPM1*-mutated AML [163]. Additionally, miR-215-5p is reported to be involved in downregulating *SMC1A* in *NPM1*-mutated AML. Expression of miR-215-5p was significantly higher, and expression of *SMC1A* was considerably lower in *NPM1*(transcript A)-mutated AML cells than in AML control cells [210].

In AML with mutated *TP53* and a complex karyotype, miR-34a is reported to be downregulated, while miR-100 is reported to be upregulated. miR-34a was correlated with resistance to chemotherapy and inferior survival. Among AML with biallelic TP53 and complex karyotype, miR-34a expression was divided into quartiles, and higher (the fourth quartile) expression of miR-34a was associated with better OS [211]. *TP53* encodes the TP53 protein, a well-known tumor suppressor. When activated, TP53 serves as a transcriptional activator for numerous genes that regulate cellular functions. Mutations in *TP53* can have various consequences. One effect is the loss of its tumour-suppressive function, which can occur due to point mutations (primarily affecting exons 5–8) or truncating variants (caused by indels) or *TP53* loss (caused by large deletions) that lead to a nonfunctional TP53 [212].

Chen et al. demonstrated that miR-363-3p can potentially trigger the *RUNX1*^mut^ AML onset [213]. Barreyro et al. showed that Hematopoietic Stem and Progenitor Cells (HSPCs) lacking miR-146a but with *RUNX1*^mut^ progresses to AML. These reports suggest a crucial role of both miR-363-3p and miR-146a in *RUNX1*^mut^ AML [214].

**Table 4:** miRNAs associated with genetic abnormalities in AML.

Genetic Abnormality	miRNA	Reference
t(8;21)(q22;q22.1)/ *RUNX1::RUNX1T1*	let-7b	[200]
	miR-223	[201]
	miR-9-1	[202]
	miR-126	[203]
	miR-130a	[204]
	miR-383	[205]
t(15;17)(q24.1;q21.2)/ *PML::RARA*	miR-15b	[206]
	miR-125b	[207]
	miR-382-5p	[208]
inv(16)(p13.1q22) or t(16;16)(p13.1;q22)/ *CBFB::MYH11*	miR-126	[169]
t(9;11)(p21.3;q23.3)/ *MLLT3::KMT2A*	miR-30e	[151]
*NPM1*	miR-10a	[209]
	miR-21	[163]
	miR-215-5p	[210]
*TP53*	miR-34a	[211]
	miR-100	
*RUNX1*	miR-363-3p	[213]
	miR-146a	[214]

## 5. Conclusions

In this review, we summarized the downregulated and upregulated miRNAs observed in AML, highlighting the presence of both types. Research has demonstrated that many miRNA expression levels are downregulated in AML. Studies have also indicated that forced upregulation of some miRNAs can inhibit the tumorigenic potential of AML cells. The downregulation and suppressive role of the miR-29 family and miR-34a are well-established in numerous studies on AML. Additionally, various reports also highlight the suppressive role of miR-142 in this context. Recent studies have suggested the suppressive role of miR-20a-5p, miR-22, miR-133, miR-135a, miR-185, miR-192, miR-199, miR-451, miR-485-5p, miR-495, miR-520a, miR-9, and miR-137; however, further investigation is needed. There have also been isolated reports on the suppressive role of miR-103a-2-5p, miR-454, miR-455-3p, and miR-148, but little is known about their functions, and further studies are required. Additionally, miR-211-5p, miR-30e-5p, miR-409-3p, miR-654-3p, and miR-1294 appear to be downregulated in AML and require further studies.

Research has reported that the expression levels of many miRNAs are elevated in AML. Studies have shown that forced downregulation of certain miRNAs can inhibit the tumorigenic potential of AML cells. The upregulation and oncogenic functions of miR-10, miR-17, miR-21, miR-155, miR-181 family, and miR-222 have been documented, alongside isolated reports on the oncogenic function of miR-1306-5p. However, these also require further research. Additional upregulated microRNAs in AML include miR-93, miR-106b-5p, miR-126, and miR-146, all of which need more research to clarify their roles in AML. Some microRNAs have been described as upregulated and downregulated; for instance, miR-92a and miR-361, suggesting that their functions remain unclear and warrant furtherer studies.

The accumulating data increasingly supports the idea that miRNA expression could significantly influence clinical outcomes in AML, and their potential is actively being explored. Many miRNAs have been correlated with patient prognosis. Higher miR-34a expression levels were associated with achieving CR [115], overexpression of miR-9 improved daunorubicin sensitivity in AML cells [178], higher miR-133 expression level was linked with longer OS and RFS [130], higher miR-135a expression was associated with better prognosis [134], and lower miR-106b-5p expression and Rab10 protein expression at the initial stage were associated with longer survival rates [193]. Conversely, diminished levels of miR-34a expression have been associated with an unfavorable prognosis in AML [115], lower miR-142-3p expression levels were associated with drug-resistant AML cell lines [137], lower miR-20 expression was correlated with shorter OS [48], higher miR-10a-5p was associated with shorter OS [185], and higher miR-21 and miR-17-5p expressions were linked to worse OS and RFS [189,190].

In particular subtypes of AML, according to ELN recommendations, we can highlight the role of some miRNAs in their leukemogenesis. miR let-7b, miR-9-1, miR-126, miR-130a, miR-223, and miR-383 seem to play an important role in AML with t(8;21)(q22;q22.1)/*RUNX1::RUNX1T1*. miR-15b, miR-125b, and miR-382-5p seem to play an essential role in APL with t(15;17)(q24.1;q21.2)/*PML::RARA*. In AML with inv(16)(p13.1q22) or t(16;16)(p13.1;q22)/*CBFB::MYH11*, miR-126 seems to play a crucial role. In AML with fusion gene *MLLT3::KMT2A*, miR-30e appears to play an important role. In *NPM1*^mut^AML, miR-10a, miR-21, and miR-215-5p appears essential. In *TP53*^mut^AML, miR-34a plays a pivotal role. In turn, in *RUNX1*^mut^AML, miR-146a and miR-363-3p may occur crucial.

Overall, the available evidence underscores the promising potential of miRNAs as novel therapeutic targets in AML. Changes in miRNA expression levels could serve as additional molecular markers for better assessment of AML patients’ prognosis, especially when combined with other molecular and cytogenetic risk factors. This review highlights many promising genetic biomarkers that could aid in AML diagnosis, prognosis, and therapy response in the future; however, further studies are necessary. The use of miRNAs in therapy still faces many challenges, such as the capability of one miRNA to regulate multiple mRNAs simultaneously, leaving a vast number of possible miRNA-mRNA interactions yet to be explored. Over the past decades, studies on miRNAs have provided valuable insights into their biological functions, roles in disease development, and methods for effectively delivering them into organisms. It is possible that in the future, miRNA expression profiles might serve as markers for measurable residual disease in AML. It is essential to note that the outcomes of miRNA expression can differ depending on the analysis platforms employed and the various AML subtypes examined, which should be considered in future research.

## Figures and Tables

**Table 1:** Characteristics of non-coding RNA (ncRNA) according to GENCODE [17].

Abbreviation/ Numbers of Nucleotides	Full Name	Main Function	Type	Number of Genes by GENCODE *	Reference
lncRNA/>200	long non-coding RNA	epigenetic, transcriptional, post-transcriptional regulation	regulatory	35,934	[18]
snRNA/75–300	small nuclear RNA	spliceosome formation, which catalyzes the splicing of pre-mRNA	housekeeping	1901	[19,20]
miRNA/19–25	microRNA	gene expression regulation at the post-transcriptional level through the destabilization of mRNA or inhibition of translation	regulatory	~2500 **	[21,22,23]
snoRNA/60–300	small nucleolar RNA	regulation of spliceosomal and ribosomal functions, maintenance of the structure of rRNA	housekeeping	942	[24,25]
tRNA/70–80	transfer RNA	protein synthesis by codon–anticodon interactions during translation	housekeeping	416 **	[26,27]
rRNA/up to~5000	ribosomal RNA	ribosome subunits formation, which take part in translation, indicating the precise positioning of ribosomal proteins within the ribosome	housekeeping	47	[28,29]
siRNA/21–23	small interfering RNA	suppression of genes expression by RNA interference (RNAi)	regulatory	N/A	[30]
circRNA/N/A	circular RNA	regulation of miRNA through the sponge effect	regulatory	~11,000 **	[31,32]
piRNA/24–32	piwi-interacting RNA	gene suppression by interactions with PIWI proteins	regulatory	~20,000 **	[33,34,35]
paRNA/200–500	promoter-associated RNA	scaffolding for proteins regulating gene expression e.g., during chromatin remodelling or transcription	regulatory	N/A	[36]
eRNA/50–2000	enhancer RNA	regulation of gene expression by modulating chromatin	regulatory	N/A	[37,38]

* Statistics about the current GENCODE Release (version 47); ** Data from article cited as a reference; N/A—not available.

## Data Availability

Not applicable.

## References

[1] Ranganathan K., Sivasankar V. (2014). MicroRNAs—Biology and clinical applications. J. Oral Maxillofac. Pathol..

[2] Metcalf G.A.D. (2024). MicroRNAs: Circulating biomarkers for the early detection of imperceptible cancers via biosensor and machine-learning advances. Oncogene.

[3] Wang J., Chen J., Sen S. (2016). MicroRNA as Biomarkers and Diagnostics. J. Cell. Physiol..

[4] Supplitt S., Karpinski P., Sasiadek M., Laczmanska I. (2021). Current Achievements and Applications of Transcriptomics in Personalized Cancer Medicine. Int. J. Mol. Sci..

[5] O’Brien J., Hayder H., Zayed Y., Peng C. (2018). Overview of MicroRNA Biogenesis, Mechanisms of Actions, and Circulation. Front. Endocrinol..

[6] Bhaskaran M., Mohan M. (2014). MicroRNAs: History, biogenesis, and their evolving role in animal development and disease. Vet. Pathol..

[7] Otmani K., Lewalle P. (2021). Tumor Suppressor miRNA in Cancer Cells and the Tumor Microenvironment: Mechanism of Deregulation and Clinical Implications. Front. Oncol..

[8] Poller W., Sahoo S., Hajjar R., Landmesser U., Krichevsky A.M. (2023). Exploration of the Noncoding Genome for Human-Specific Therapeutic Targets-Recent Insights at Molecular and Cellular Level. Cells.

[9] Walter N.G. (2024). Are non-protein coding RNAs junk or treasure?: An attempt to explain and reconcile opposing viewpoints of whether the human genome is mostly transcribed into non-functional or functional RNAs. Bioessays.

[10] Kelly R.C., Morgan R.A., Brown M., Overton I., Hardiman G. (2024). The Non-coding Genome and Network Biology. Systems Biology II Springer Medizin.

[11] Nobusada T., Yip C.W., Agrawal S., Severin J., Abugessaisa I., Hasegawa A., Hon C.C., Ide S., Koido M., Kondo A. (2025). Update of the FANTOM web resource: Enhancement for studying noncoding genomes. Nucleic Acids Res..

[12] Loganathan T., Doss G.P.C. (2023). Non-coding RNAs in human health and disease: Potential function as biomarkers and therapeutic targets. Funct. Integr. Genom..

[13] George T.P., Subramanian S., Supriya M.H. (2024). A brief review of noncoding RNA. Egypt. J. Med. Hum. Genet..

[14] Wang Z., Wang H., Zhou S., Mao J., Zhan Z., Duan S. (2024). miRNA interplay: Mechanisms and therapeutic interventions in cancer. MedComm—Oncol..

[15] Poliseno L., Lanza M., Pandolfi P.P. (2024). Coding, or non-coding, that is the question. Cell Res..

[16] Li S., Hu W., Qian L., Sun D. (2025). Insights into non-coding RNAS: Biogenesis, function and their potential regulatory roles in acute kidney disease and chronic kidney disease. Mol. Cell. Biochem..

[17] GENCODE https://www.gencodegenes.org/human/stats.html.

[18] Malgundkar S.H., Tamimi Y. (2024). The pivotal role of long non-coding RNAs as potential biomarkers and modulators of chemoresistance in ovarian cancer (OC). Hum. Genet..

[19] Kasprzyk M.E., Kazimierska M., Podralska M. (2023). Navigating Non-Coding RNA from Biogenesis to Therapeutic Application.

[20] Su Y., Wu J., Chen W., Shan J., Chen D., Zhu G., Ge S., Liu Y. (2024). Spliceosomal snRNAs, the Essential Players in pre-mRNA Processing in Eukaryotic Nucleus: From Biogenesis to Functions and Spatiotemporal Characteristics. Adv. Biol..

[21] Wang H., Liu J., Fang Y., Shen X., Liu H., Yu L., Zeng S., Cai S., Zhou J., Li Z. (2024). Design and analysis of self-priming extension DNA hairpin probe for miRNA detection based on a unified dynamic programming framework. Anal. Chim. Acta.

[22] Venneri M., Passantino A. (2023). MiRNA: What clinicians need to know. Eur. J. Intern. Med..

[23] Maji R.K., Leisegang M.S., Boon R.A., Schulz M.H. (2025). Revealing microRNA regulation in single cells. Trends Genet..

[24] Chauhan W., Sudharshan S.J., Kafle S., Zennadi R. (2024). SnoRNAs: Exploring Their Implication in Human Diseases. Int. J. Mol. Sci..

[25] Shen L.P., Zhang W.C., Deng J.R., Qi Z.H., Lin Z.W., Wang Z.D. (2024). Advances in the mechanism of small nucleolar RNA and its role in DNA damage response. Mil. Med. Res..

[26] Smith T.J., Giles R.N., Koutmou K.S. (2024). Anticodon stem-loop tRNA modifications influence codon decoding and frame maintenance during translation. Semin. Cell Dev. Biol..

[27] Wang L., Lin S. (2023). Emerging functions of tRNA modifications in mRNA translation and diseases. J. Genet. Genom..

[28] News Medical Life Sciences. https://www.news-medical.net/life-sciences/-Types-of-RNA-mRNA-rRNA-and-tRNA.aspx#:~:text=In%20bacteria%2C%20the%20small%20and,1800%20and%205000%20nucleotides%2C%20respectively.

[29] Rauscher R., Polacek N. (2024). Ribosomal RNA expansion segments and their role in ribosome biology. Biochem. Soc. Trans..

[30] Li R., Zhu M., Hu X., Chen J., Yu F., Barth S., Sun L., He H. (2024). Overcoming endosomal/lysosomal barriers: Advanced strategies for cytosolic siRNA delivery. Chin. Chem. Lett..

[31] Saleem A., Khan M.U., Zahid T., Khurram I., Ghani M.U., Ullah I., Munir R., Calina D., Sharifi-Rad J. (2024). Biological role and regulation of circular RNA as an emerging biomarker and potential therapeutic target for cancer. Mol. Biol. Rep..

[32] Drula R., Braicu C., Neagoe I.B. (2024). Current advances in circular RNA detection and investigation methods: Are we running in circles?. Wiley Interdiscip. Rev.-RNA.

[33] Werry N., Russell S.J., Sivakumar R., Miller S., Hickey K., Larmer S., Lohuis M., Librach C., LaMarre J. (2024). piRNA expression patterns in high vs. low fertility bovine sperm. Syst. Biol. Reprod. Med..

[34] Claro-Linares F., Rojas-Ríos P. (2025). PIWI proteins and piRNAs: Key regulators of stem cell biology. Front. Cell Dev. Biol..

[35] Jove https://app.jove.com/science-education/v/11630/concepts/pirna-piwi-interacting-rnas.

[36] Márton É., Varga A., Domoszlai D., Buglyó G., Balázs A., Penyige A., Balogh I., Nagy B., Szilágyi M. (2025). Non-Coding RNAs in Cancer: Structure, Function, and Clinical Application. Cancers.

[37] Colino-Sanguino Y., Clark S.J., Valdes-Mora F. (2022). The H2A.Z-nucleosome code in mammals: Emerging functions. Trends Genet..

[38] Ma S., Wang Z., Xiong Z., Ge Y., Xu M.-Y., Zhang J., Peng Y., Zhang Q., Sun J., Xi Z. (2025). Enhancer transcription profiling reveals an enhancer RNA-driven ferroptosis and new therapeutic opportunities in prostate cancer. Signal Transduct. Target. Ther..

[39] Rodriguez A., Griffiths-Jones S., Ashurst J.L., Bradley A. (2004). Identification of mammalian microRNA host genes and transcription units. Genome Res..

[40] Yin Z., Shen H., Gu C.M., Zhang M.Q., Liu Z., Huang J., Zhu Y., Zhong Q., Huang Y., Wu F. (2021). MiRNA-142-3P and FUS can be Sponged by Long Noncoding RNA DUBR to Promote Cell Proliferation in Acute Myeloid Leukemia. Front. Mol. Biosci..

[41] Orang A.V., Safaralizadeh R., Kazemzadeh-Bavili M. (2014). Mechanisms of miRNA-Mediated Gene Regulation from Common Downregulation to mRNA-Specific Upregulation. Int. J. Genom..

[42] Machowska M., Galka-Marciniak P., Kozlowski P. (2022). Consequences of genetic variants in miRNA genes. Comput. Struct. Biotechnol. J..

[43] Lee R.C., Feinbaum R.L., Ambros V. (1993). The *C. elegans* heterochronic gene lin-4 encodes small RNAs with antisense complementarity to lin-14. Cell.

[44] Liu B., Shyr Y., Cai J., Liu Q. (2018). Interplay between miRNAs and host genes and their role in cancer. Brief. Funct. Genom..

[45] Vilimova M., Pfeffer S. (2023). Post-transcriptional regulation of polycistronic microRNAs. Wiley Interdiscip. Rev..

[46] Le T.A.H., Lao T.D. (2022). Circulating microRNAs as the Potential Diagnostic and Prognostic Biomarkers for Nasopharyngeal Carcinoma. Genes.

[47] Santovito D., Weber C. (2022). Non-canonical features of microRNAs: Paradigms emerging from cardiovascular disease. Nat. Rev. Cardiol..

[48] Macfarlane L.A., Murphy P.R. (2010). MicroRNA: Biogenesis, Function and Role in Cancer. Curr. Genom..

[49] Shademan B., Karamad V., Nourazarian A., Masjedi S., Isazadeh A., Sogutlu F., Avci C.B. (2023). MicroRNAs as Targets for Cancer Diagnosis: Interests and Limitations. Adv. Pharm. Bull..

[50] Cai X., Hagedorn C.H., Cullen B.R. (2004). Human microRNAs are processed from capped, polyadenylated transcripts that can also function as mRNAs. RNA.

[51] Lee D., Shin C. (2018). Emerging roles of DROSHA beyond primary microRNA processing. RNA Biol..

[52] Han J., Pedersen J.S., Kwon S.C., Belair C.D., Kim Y.K., Yeom K.H., Yang W.Y., Haussler D., Blelloch R., Kim V.N. (2009). Posttranscriptional crossregulation between Drosha and DGCR8. Cell.

[53] Kwon S.C., Nguyen T.A., Choi Y.G., Jo M.H., Hohng S., Kim V.N., Woo J.S. (2016). Structure of Human DROSHA. Cell.

[54] Bofill-De Ros X., Vang Ørom U.A. (2024). Recent progress in miRNA biogenesis and decay. RNA Biol..

[55] Stavast C.J., Erkeland S.J. (2019). The Non-Canonical Aspects of MicroRNAs: Many Roads to Gene Regulation. Cells.

[56] Weng Y.T., Chang Y.M., Chern Y. (2023). The Impact of Dysregulated microRNA Biogenesis Machinery and microRNA Sorting on Neurodegenerative Diseases. Int. J. Mol. Sci..

[57] Michlewski G., Cáceres J.F. (2019). Post-transcriptional control of miRNA biogenesis. RNA.

[58] Nakanishi K. (2024). When Argonaute takes out the ribonuclease sword. J. Biol. Chem..

[59] Grenda A., Budzyński M., Filip A.A. (2013). Biogenesis of microRNAs and their role in the development and course of selected hematologic disorders. Postępy Hig. Med. Doświadczalnej Online.

[60] Hammond S.M. (2015). An overview of microRNAs. Adv. Drug Deliv. Rev..

[61] Jungers C.F., Djuranovic S. (2022). Modulation of miRISC-Mediated Gene Silencing in Eukaryotes. Front. Mol. Biosci..

[62] Kehl T., Backes C., Kern F., Fehlmann T., Ludwig N., Meese E., Lenhof H.P., Keller A. (2017). About miRNAs, miRNA seeds, target genes and target pathways. Oncotarget.

[63] Li L., Sheng P., Li T., Fields C.J., Hiers N.M., Wang Y., Li J., Guardia C.M., Licht J.D., Xie M. (2021). Widespread microRNA degradation elements in target mRNAs can assist the encoded proteins. Genes Dev..

[64] Hackl L.M., Fenn A., Louadi Z., Baumbach J., Kacprowski T., List M., Tsoy O. (2023). Alternative splicing impacts microRNA regulation within coding regions. NAR Genom. Bioinform..

[65] Naeli P., Winter T., Hackett A.P., Alboushi L., Jafarnejad S.M. (2023). The intricate balance between microRNA-induced mRNA decay and translational repression. FEBS J..

[66] Kuzuoğlu-Öztürk D., Bhandari D., Huntzinger E., Fauser M., Helms S., Izaurralde E. (2016). miRISC and the CCR4-NOT complex silence mRNA targets independently of 43S ribosomal scanning. EMBO J..

[67] Amorim I.S., Lach G., Gkogkas C.G. (2018). The Role of the Eukaryotic Translation Initiation Factor 4E (eIF4E) in Neuropsychiatric Disorders. Front. Genet..

[68] Eulalio A., Huntzinger E., Nishihara T., Rehwinkel J., Fauser M., Izaurralde E. (2009). Deadenylation is a widespread effect of miRNA regulation. RNA.

[69] Arber D.A., Orazi A., Hasserjian R.P., Borowitz M.J., Calvo K.R., Kvasnicka H.-M., Wang S.A., Bagg A., Barbui T., Branford S. (2022). International Consensus Classification of Myeloid Neoplasms and Acute Leukemias: Integrating morphologic, clinical, and genomic data. Blood.

[70] Grove C.S., Vassiliou G.S. (2014). Acute myeloid leukemia: A paradigm for the clonal evolution of cancer?. Dis. Models Mech..

[71] American Cancer Society https://www.cancer.org/cancer/types/acute-myeloid-leukemia/causes-risks-prevention/what-causes.html.

[72] American Cancer Society https://www.cancer.org/cancer/types/acute-myeloid-leukemia/treating.html.

[73] Jimenez-Chillon C., Dillon R., Russell N. (2024). Optimal Post-Remission Consolidation Therapy in Patients with AML. Acta Haematol. Pol..

[74] Abuelgasim K.A., Albuhayri B., Munshi R., Mugairi A.A., Alahmari B., Gmati G., Salama H., Alzahrani M., Alhejazi A., Alaskar A. (2020). Impact of age and induction therapy on outcome of 180 adult patients with acute myeloid leukemia; retrospective analysis and literature review. Leuk. Res. Rep..

[75] National Cancer Institute https://seer.cancer.gov/statfacts/html/amyl.html.

[76] Peng Y., Croce C.M. (2016). The role of MicroRNAs in human cancer. Signal Transduct. Target. Ther..

[77] Wallace J.A., O’Connell R.M. (2017). MicroRNAs and acute myeloid leukemia: Therapeutic implications and emerging concepts. Blood.

[78] Fletcher D., Brown E., Javadala J., Uysal-Onganer P., Guinn B.A. (2022). microRNA expression in acute myeloid leukemia: New targets for therapy?. eJHaem.

[79] Kim T., Croce C.M. (2023). MicroRNA: Trends in clinical trials of cancer diagnosis and therapy strategies. Exp. Mol. Med..

[80] Target Scan Human https://www.targetscan.org/vert_80/.

[81] miRDB https://mirdb.org/.

[82] ENCORI https://rnasysu.com/encori/.

[83] Ramsingh G., Jacoby M.A., Shao J., De Jesus Pizzaro R.E., Shen D., Trissal M., Getz A.H., Ley T.J., Walter M.J., Link D.C. (2013). Acquired copy number alterations of miRNA genes in acute myeloid leukemia are uncommon. Blood.

[84] Starczynowski D.T., Morin R., McPherson A., Lam J., Chari R., Wegrzyn J., Kuchenbauer F., Hirst M., Tohyama K., Humphries R.K. (2011). Genome-wide identification of human microRNAs located in leukemia-associated genomic alterations. Blood.

[85] Eyholzer M., Schmid S., Wilkens L., Mueller B.U., Pabst T. (2010). The tumor-suppressive miR-29a/b1 cluster is regulated by CEBPA and blocked in human AML. Br. J. Cancer.

[86] Li W., Wang Y., Liu R., Kasinski A.L., Shen H., Slack F.J., Tang D.G. (2021). MicroRNA-34a: Potent Tumor Suppressor, Cancer Stem Cell Inhibitor, and Potential Anticancer Therapeutic. Front. Cell Dev. Biol..

[87] Bousquet M., Quelen C., Rosati R., Mansat-De Mas V., La Starza R., Bastard C., Lippert E., Talmant P., Lafage-Pochitaloff M., Leroux D. (2008). Myeloid cell differentiation arrest by miR-125b-1 in myelodysplastic syndrome and acute myeloid leukemia with the t(2;11)(p21;q23) translocation. J. Exp. Med..

[88] Mi S., Li Z., Chen P., He C., Cao D., Elkahloun A., Lu J., Pelloso L.A., Wunderlich M., Huang H. (2010). Aberrant overexpression and function of the miR-17-92 cluster in MLL-rearranged acute leukemia. Proc. Natl. Acad. Sci. USA.

[89] Chen P., Price C., Li Z., Li Y., Cao D., Wiley A., He C., Gurbuxani S., Kunjamma R.B., Huang H. (2013). miR-9 is an essential oncogenic microRNA specifically overexpressed in mixed lineage leukemia-rearranged leukemia. Proc. Natl. Acad. Sci. USA.

[90] Senyuk V., Zhang Y., Liu Y., Ming M., Premanand K., Zhou L., Chen P., Chen J., Rowley J.D., Nucifora G. (2013). Critical role of miR-9 in myelopoiesis and EVI1-induced leukemogenesis. Proc. Natl. Acad. Sci. USA.

[91] Li Z., Lu J., Sun M., Mi S., Zhang H., Luo R.T., Chen P., Wang Y., Yan M., Qian Z. (2008). Distinct microRNA expression profiles in acute myeloid leukemia with common translocations. Proc. Natl. Acad. Sci. USA.

[92] Gao X.N., Lin J., Li Y.H., Gao L., Wang X.R., Wang W., Kang H.Y., Yan G.T., Wang L.L., Yu L. (2011). MicroRNA-193a represses c-kit expression and functions as a methylation-silenced tumor suppressor in acute myeloid leukemia. Oncogene.

[93] Pulikkan J.A., Dengler V., Peramangalam P.S., Peer Zada A.A., Müller-Tidow C., Bohlander S.K., Tenen D.G., Behre G. (2010). Cell-cycle regulator E2F1 and microRNA-223 comprise an autoregulatory negative feedback loop in acute myeloid leukemia. Blood.

[94] Vineetha R.C., Raj J.A.G., Devipriya P., Mahitha M.S., Hariharan S. (2024). MicroRNA-based therapies: Revolutionizing the treatment of acute myeloid leukemia. Int. J. Lab. Hematol..

[95] Ghazaryan A., Wallace J.A., Tang W.W., Barba C., Lee S.-H., Bauer K.M., Nelson M.C., Kim C.N., Stubben C., Voth W.P. (2023). miRNA-1 promotes acute myeloid leukemia cell pathogenesis through metabolic regulation. Front. Genet..

[96] Salehi A. (2024). A novel therapeutic strategy: The significance of exosomal miRNAs in acute myeloid leukemia. Med. Oncol..

[97] Iacomino G. (2023). miRNAs: The Road from Bench to Bedside. Genes.

[98] Wang C., Li L., Li M., Wang W., Liu Y., Wang S. (2020). Silencing long non-coding RNA XIST suppresses drug resistance in acute myeloid leukemia through down-regulation of MYC by elevating microRNA-29a expression. Mol. Med..

[99] Tang Y.-J., Wu W., Chen Q.-Q., Liu S.-H., Zheng Z.-Y., Cui Z.-L., Xu J.-P., Xue Y., Lin D.-H. (2022). miR-29b-3p suppresses the malignant biological behaviors of AML cells via inhibiting NF-κB and JAK/STAT signaling pathways by targeting HuR. BMC Cancer.

[100] Randazzo V., Salemi D., Agueli C., Cannella S., Marfia A., Bica M.G., Randazzo G., Russo Lacerna C., Di Raimondo F., Fabbiano F. (2016). Upregulation of Mir-29 in Normal Karyotype Aml Showing Dnmt3a Mutation. J. Hematol. Transfus..

[101] Ngankeu A., Ranganathan P., Havelange V., Nicolet D., Volinia S., Powell B.L., Kolitz J.E., Uy G.L., Stone R.M., Kornblau S.M. (2017). Discovery and functional implications of a miR-29b-1/miR-29a cluster polymorphism in acute myeloid leukemia. Oncotarget.

[102] Abdellateif M.S., Hassan N.M., Kamel M.M., El-Meligui Y.M. (2024). Bone marrow microRNA-34a is a good indicator for response to treatment in acute myeloid leukemia. Oncol. Res..

[103] Wang X., Li J., Dong K., Lin F., Long M., Ouyang Y., Wei J., Chen X., Weng Y., He T. (2015). Tumor suppressor miR-34a targets PD-L1 and functions as a potential immunotherapeutic target in acute myeloid leukemia. Cell. Signal..

[104] Hu Y., Ma X., Wu Z., Nong Q., Liu F., Wang Y., Dong M. (2020). MicroRNA-34a-mediated death of acute myeloid leukemia stem cells through apoptosis induction and exosome shedding inhibition via histone deacetylase 2 targeting. IUBMB Life.

[105] Huang Y., Zou Y., Lin L., Ma X., Chen H. (2018). Identification of serum miR-34a as a potential biomarker in acute myeloid leukemia. Cancer Biomark..

[106] Wen J., Chen Y., Liao C., Ma X., Wang M., Li Q., Wang D., Li Y., Zhang X., Li L. (2023). Engineered mesenchymal stem cell exosomes loaded with miR-34c-5p selectively promote eradication of acute myeloid leukemia stem cells. Cancer Lett..

[107] Peng D., Wang H., Li L., Ma X., Chen Y., Zhou H., Luo Y., Xiao Y., Liu L. (2018). miR-34c-5p promotes eradication of acute myeloid leukemia stem cells by inducing senescence through selective RAB27B targeting to inhibit exosome shedding. Leukemia.

[108] Yang D.-Q., Zhou J.-D., Wang Y.-X., Deng Z.-Q., Yang J., Yao D.-M., Qian Z., Yang L., Lin J., Qian J. (2017). Low miR-34c expression is associated with poor outcome in de novo acute myeloid leukemia. Int. J. Lab. Hematol..

[109] Hong D.S., Kang Y.-K., Borad M., Sachdev J., Ejadi S., Lim H.Y., Brenner A.J., Park K., Lee J.-L., Kim T.-Y. (2020). Phase 1 study of MRX34, a liposomal miR-34a mimic, in patients with advanced solid tumors. Br. J. Cancer.

[110] Zhang Y., Liu Y., Xu X. (2017). Upregulation of miR-142-3p Improves Drug Sensitivity of Acute Myelogenous Leukemia through Reducing P-Glycoprotein and Repressing Autophagy by Targeting HMGB1. Transl. Oncol..

[111] Jiang Z., Liu T., Wang Y., Li J., Guo L. (2024). Effect of lncRNA XIST on acute myeloid leukemia cells via miR-142-5p-PFKP axis. Hematology.

[112] Yuan D.M., Ma J., Fang W.B. (2019). Identification of non-coding RNA regulatory networks in pediatric acute myeloid leukemia reveals circ-0004136 could promote cell proliferation by sponging miR-142. Eur. Rev. Med. Pharmacol. Sci..

[113] Zhang B., Zhao D., Chen F., Frankhouser D., Wang H., Pathak K.V., Dong L., Torres A., Garcia-Mansfield K., Zhang Y. (2023). Acquired miR-142 deficit in leukemic stem cells suffices to drive chronic myeloid leukemia into blast crisis. Nat. Commun..

[114] Liu Y., Lei P., Qiao H., Sun K., Lu X., Bao F., Yu R., Lian C., Li Y., Chen W. (2019). miR-9 Enhances the Chemosensitivity of AML Cells to Daunorubicin by Targeting the EIF5A2/MCL-1 Axis. Int. J. Biol. Sci..

[115] Wang G., Yu X., Xia J., Sun J., Huang H., Liu Y. (2021). MicroRNA-9 restrains the sharp increase and boost apoptosis of human acute myeloid leukemia cells by adjusting the Hippo/YAP signaling pathway. Bioengineered.

[116] Zhu B., Xi X., Liu Q., Cheng Y., Yang H. (2019). MiR-9 functions as a tumor suppressor in acute myeloid leukemia by targeting CX chemokine receptor 4. Am. J. Transl. Res..

[117] Bao F., Zhang L., Pei X., Lian C., Liu Y., Tan H., Lei P. (2021). MiR-20a-5p functions as a potent tumor suppressor by targeting PPP6C in acute myeloid leukemia. PLoS ONE.

[118] Ping L., Jian-Jun C., Chu-Shu L., Guang-Hua L., Ming Z. (2019). Silencing of circ_0009910 inhibits acute myeloid leukemia cell growth through increasing miR-20a-5p. Blood Cells Mol. Dis..

[119] Chen Z.-H., Wang W.-T., Huang W., Fang K., Sun Y.-M., Liu S.-R., Luo X.-Q., Chen Y.-Q. (2017). The lncRNA HOTAIRM1 regulates the degradation of PML-RARA oncoprotein and myeloid cell differentiation by enhancing the autophagy pathway. Cell Death Differ..

[120] Cen Q., Chen J., Guo J., Chen M., Wang H., Wu S., Zhang H., Xie X., Li Y. (2024). CLPs-miR-103a-2-5p inhibits proliferation and promotes cell apoptosis in AML cells by targeting LILRB3 and Nrf2/HO-1 axis, regulating CD8+ T cell response. J. Transl. Med..

[121] Zheng Z.-Z., Ma Y.-P., Wu R.-H., Rong G., Li C., Li G.-X., Ren F.-G., Xu L.-J. (2020). Serum miR-133 as a novel biomarker for predicting treatment response and survival in acute myeloid leukemia. Eur. Rev. Med. Pharmacol. Sci..

[122] Yamamoto H., Lu J., Oba S., Kawamata T., Yoshimi A., Kurosaki N., Yokoyama K., Matsushita H., Kurokawa M., Tojo A. (2016). miR-133 regulates Evi1 expression in AML cells as a potential therapeutic target. Sci. Rep..

[123] Wang Q., Yue C., Liu Q., Che X. (2022). Exploration of differentially expressed mRNAs and miRNAs for pediatric acute myeloid leukemia. Front. Genet..

[124] Xu H., Wen Q. (2018). Downregulation of miR-135a predicts poor prognosis in acute myeloid leukemia and regulates leukemia progression via modulating HOXA10 expression. Mol. Med. Rep..

[125] Cheng Y.C., Fan Z., Liang C., Peng C.J., Li Y., Wang L.N., Luo J.S., Zhang X.L., Liu Y., Zhang L.D. (2024). miR-133a and miR-135a Regulate All-Trans Retinoic Acid-Mediated Differentiation in Pediatric Acute Myeloid Leukemia by Inhibiting CDX2 Translation and Serve as Prognostic Biomarkers. Technol. Cancer Res. Treat..

[126] Liu L., Yu K., Yu J., Tao W., Wei Y. (2024). MiR-133 promotes the multidrug resistance of acute myeloid leukemia cells (HL-60/ADR) to daunorubicin. Cytotechnology.

[127] Hu Y., Dong X., Chu G., Lai G., Zhang B., Wang L., Zhao Y. (2017). miR-137 downregulates c-kit expression in acute myeloid leukemia. Leuk. Res..

[128] Wang S., Zhang B.S., Yang Y., Li Y., Lv J.L., Cheng Y. (2020). TRIM25 contributes to the malignancy of acute myeloid leukemia and is negatively regulated by microRNA-137. Open Med..

[129] Wang X.-X., Zhang R., Li Y. (2017). Expression of the miR-148/152 Family in Acute Myeloid Leukemia and its Clinical Significance. Med. Sci. Monit..

[130] Wang X.-X., Zhang H., Li Y. (2019). Preliminary study on the role of miR-148a and DNMT1 in the pathogenesis of acute myeloid leukemia. Mol. Med. Rep..

[131] Pang B., Mao H., Wang J., Yang W. (2022). MiR-185-5p suppresses acute myeloid leukemia by inhibiting GPX1. Microvasc. Res..

[132] Zhang W., Liu Y., Zhang J., Zheng N. (2020). Long Non-Coding RNA Taurine Upregulated Gene 1 Targets miR-185 to Regulate Cell Proliferation and Glycolysis in Acute Myeloid Leukemia Cells in vitro. OncoTargets Ther..

[133] Wu W., Deng J., Chen C., Ma X., Yu L., Chen L. (2023). Circ_0001602 aggravates the progression of acute myeloid leukemia by regulating the miR-192-5p/ZBTB20 axis. Hematology.

[134] Tian C., Zhang L., Li X., Zhang Y., Li J., Chen L. (2018). Low miR-192 expression predicts poor prognosis in pediatric acute myeloid leukemia. Cancer Biomark..

[135] Ke S., Li R.C., Lu J., Meng F.K., Feng Y.K., Fang M.H. (2017). MicroRNA-192 regulates cell proliferation and cell cycle transition in acute myeloid leukemia via interaction with CCNT2. Int. J. Hematol..

[136] Chen D.P., Chang S.W., Wen Y.H., Wang W.T. (2023). Association between diminished miRNA expression and the disease status of AML patients: Comparing to healthy control. Biomed. J..

[137] Krakowsky R.H.E., Wurm A.A., Gerloff D., Katzerke C., Bräuer-Hartmann D., Hartmann J.U., Wilke F., Thiede C., Müller-Tidow C., Niederwieser D. (2018). miR-451a abrogates treatment resistance in FLT3-ITD-positive acute myeloid leukemia. Blood Cancer J..

[138] Li L., Mussack V., Görgens A., Pepeldjiyska E., Hartz A.S., Aslan H., Rackl E., Rank A., Schmohl J., El Andaloussi S. (2023). The potential role of serum extracellular vesicle derived small RNAs in AML research as non-invasive biomarker. Nanoscale Adv..

[139] Song L., Lin H.-S., Gong J.-N., Han H., Wang X.-S., Su R., Chen M.-T., Shen C., Ma Y.-N., Yu J. (2017). microRNA-451-modulated hnRNP A1 takes a part in granulocytic differentiation regulation and acute myeloid leukemia. Oncotarget.

[140] Su R., Gong J.-N., Chen M.-T., Song L., Shen C., Zhang X.-H., Yin X.-L., Ning H.-M., Liu B., Wang F. (2016). c-Myc suppresses miR-451⊣YWTAZ/AKT axis via recruiting HDAC3 in acute myeloid leukemia. Oncotarget.

[141] Wu K., Li Y., Nie B., Guo C., Ma X., Li L., Cheng S., Li Y., Luo S., Zeng Y. (2024). MEF2A is a transcription factor for circPVT1 and contributes to the malignancy of acute myeloid leukemia. Int. J. Oncol..

[142] Xie Y., Tan L., Wu K., Li D., Li C. (2023). MiR-455-3p mediates PPARα through UBN2 to promote apoptosis and autophagy in acute myeloid leukemia cells. Exp. Hematol..

[143] Zhang F., Li Q., Zhu K., Zhu J., Li J., Yuan Y., Zhang P., Zhou L., Liu L. (2020). LncRNA LINC00265/miR-485-5p/IRF2-mediated autophagy suppresses apoptosis in acute myeloid leukemia cells. Am. J. Transl. Res..

[144] Wang W.-L., Wang H.R., Ji W.G., Guo S.L., Li H.X., Xu X.Y. (2019). MiRNA-485-5p suppresses the proliferation of acute myeloid leukemia via targeting SALL4. Eur. Rev. Med. Pharmacol. Sci..

[145] Huang L., Dai J. (2022). Expression and Clinical Significance of miRNA-495 in the Peripheral Blood of Acute Myeloid Leukemia Patients. Proc. Anticancer Res..

[146] Lei Y., Li X., Zhu L. (2024). Matrine regulates miR-495-3p/miR-543/PDK1 axis to repress the progression of acute myeloid leukemia via the Wnt/βcatenin pathway. Chem. Biol. Drug Des..

[147] Wang G., Li X., Song L., Pan H., Jiang J., Sun L. (2019). Long noncoding RNA MIAT promotes the progression of acute myeloid leukemia by negatively regulating miR-495. Leuk. Res..

[148] Zhang W., Wan B., Liu B., Wu S., Zhao L. (2020). Clinical significance of miR-372 and miR-495 in acute myeloid leukemia. Oncol. Lett..

[149] Chen X.Y., Qin X.H., Xie X.L., Liao C.X., Liu D.T., Li G.W. (2022). Overexpression miR-520a-3p inhibits acute myeloid leukemia progression via targeting MUC1. Transl. Oncol..

[150] Xiao J., Wan F., Tian L., Li Y. (2024). Tumor suppressor miR-520a inhibits cell growth by negatively regulating PI3K/AKT signaling pathway in acute myeloid leukemia. Adv. Clin. Exp. Med..

[151] Ge Y., Hong M., Zhang Y., Wang J., Li L., Zhu H., Sheng Y., Wu W.-S., Zhang Z. (2024). miR-30e-5p regulates leukemia stem cell self-renewal through the Cyb561/ROS signaling pathway. Haematologica.

[152] Ye Q., Ren L., Jiang Z.M., Li X.Y., Wei G.Y., Ren Y.F., Ren L.H. (2023). Cryptanshinone extract of Salvia miltiorrhiza stimulates pediatric acute myeloid leukemia stem cell apoptosis and the anti-inflammatory mechanism via accelerating microRNA-211-5p to supress Janus kinase 2/signal transducer and activator of transcription 3 signaling pathway activation. J. Physiol. Pharmacol..

[153] Xie W., Wang Z., Guo X., Guan H. (2023). MiR-409-3p regulates the proliferation and apoptosis of THP-1 through targeting Rab10. Leuk. Res..

[154] Wang X., Zhong L., Dan W., Chu X., Luo X., Liu C., Wan P., Lu Y., Liu Z., Zhang Z. (2023). MiR-454-3p promotes apoptosis and autophagy of AML cells by targeting ZEB2 and regulating AKT/mTOR pathway. Hematology.

[155] Wang J., Wu C., Zhou W. (2023). CircPLXNB2 regulates acute myeloid leukemia progression through miR-654-3p/CCND1 axis. Hematology.

[156] Wang S., Zhang B.S., Yang Y., Fu L.L. (2024). CircFN1 promotes acute myeloid leukemia cell proliferation and invasion but refrains apoptosis via miR-1294/ARHGEF10L axis. Kaohsiung J. Med. Sci..

[157] Bi L., Sun L., Jin Z., Zhang S., Shen Z. (2018). MicroRNA-10a/b are regulators of myeloid differentiation and acute myeloid leukemia. Oncol. Lett..

[158] Wang C.J., Zou H., Feng G.F. (2018). MiR-10b regulates the proliferation and apoptosis of pediatric acute myeloid leukemia through targeting HOXD10. Eur. Rev. Med. Pharmacol. Sci..

[159] Zhi Y., Xie X., Wang R., Wang B., Gu W., Ling Y., Dong W., Zhi F., Liu Y. (2015). Serum level of miR-10-5p as a prognostic biomarker for acute myeloid leukemia. Int. J. Hematol..

[160] Yuan Z., Wang W. (2020). LncRNA SNHG4 regulates miR-10a/PTEN to inhibit the proliferation of acute myeloid leukemia cells. Hematology.

[161] Li C., Yan H., Yin J., Ma J., Liao A., Yang S., Wang L., Huang Y., Lin C., Dong Z. (2019). MicroRNA-21 promotes proliferation in acute myeloid leukemia by targeting Krüppel-like factor 5. Oncol. Lett..

[162] Li X., Zhang X., Ma H., Liu Y., Cheng S., Wang H., Sun J. (2022). Upregulation of serum exosomal miR-21 was associated with poor prognosis of acute myeloid leukemia patients. Food Sci. Technol..

[163] Riccioni R., Lulli V., Castelli G., Biffoni M., Tiberio R., Pelosi E., Lo-Coco F., Testa U. (2015). miR-21 is overexpressed in NPM1-mutant acute myeloid leukemias. Leuk. Res..

[164] Cao Y., Liu Y., Shang L., Chen H., Yue Y., Dong W., Guo Y., Yang H., Yang X., Liu Y. (2022). Overexpression of miR-17 predicts adverse prognosis and disease recurrence for acute myeloid leukemia. Int. J. Clin. Oncol..

[165] Liu M., Yu B., Tian Y., Li F. (2024). Regulatory function and mechanism research for m6A modification WTAP via SUCLG2-AS1- miR-17-5p-JAK1 axis in AML. BMC Cancer.

[166] Wang W., Liu J., Chen K., Wang J., Dong Q., Xie J., Yuan Y. (2021). Vitamin D promotes autophagy in AML cells by inhibiting miR-17-5p-induced Beclin-1 overexpression. Mol. Cell. Biochem..

[167] Huang J., Xiao R., Wang X., Khadka B., Fang Z., Yu M., Zhang L., Wu J., Liu J. (2021). MicroRNA-93 knockdown inhibits acute myeloid leukemia cell growth via inactivating the PI3K/AKT pathway by upregulating DAB2. Int. J. Oncol..

[168] Xu L., Wang A., Guan H. (2024). microRNA-106b-5p and Rab10: Potential Markers of Acute Myeloid Leukemia. Cancer Biother Radiopharm.

[169] Zhang L., Nguyen L.X.T., Chen Y.C., Wu D., Cook G.J., Hoang D.H., Brewer C.J., He X., Dong H., Li S. (2021). Targeting miR-126 in inv(16) acute myeloid leukemia inhibits leukemia development and leukemia stem cell maintenance. Nat. Commun..

[170] Wang L., Zhang H., Lei D. (2019). microRNA-146a Promotes Growth of Acute Leukemia Cells by Downregulating Ciliary Neurotrophic Factor Receptor and Activating JAK2/STAT3 Signaling. Yonsei Med. J..

[171] Li X., Xu L., Sheng X., Cai J., Liu J., Yin T., Xiao F., Chen F., Zhong H. (2018). Upregulated microRNA-146a expression induced by granulocyte colony-stimulating factor enhanced low-dosage chemotherapy response in aged acute myeloid leukemia patients. Exp. Hematol..

[172] Garavand J., Mohammadi M.H., Jalali M.T., Saki N. (2024). Correlation of miR-155-5p, KRAS, and CREB Expression in Patients with Acute Myeloid Leukemia. Clin. Lab..

[173] Xue H., Hua L.M., Guo M., Luo J.M. (2014). SHIP1 is targeted by miR-155 in acute myeloid leukemia. Oncol. Rep..

[174] Palma C.A., Al Sheikha D., Lim T.K., Bryant A., Vu T.T., Jayaswal V., Ma D.D. (2014). MicroRNA-155 as an inducer of apoptosis and cell differentiation in Acute Myeloid Leukemia. Mol. Cancer.

[175] Elgohary T., Abu-Taleb F., Ghonaim R. (2019). The Impact of miRNA-155 Expression on Treatment Outcome in Adult Acute Myeloid Leukemia Patients. J. Cancer Ther..

[176] Hatem A., Gab~Allah A., Ghonaim R., Haggag R. (2021). Prognostic Impact of microRNAs (miR-155, miR-10a, let7a) on the Outcome of Adult Patients with Acute Myeloid Leukemia. Zagazig Univ. Med. J..

[177] El-Hassib D.M.A., Zidan M.A.-A., Marei Y.M., El Gheit N.E.S.N.A., Alnoury H.A. (2023). Study of Micro RNA 181 a3p As a Biomarker for Diagnosis of Acute Myeloid Leukemia. Egypt. J. Hosp. Med..

[178] Butrym A., Rybka J., Baczyńska D., Poręba R., Mazur G., Kuliczkowski K. (2016). Expression of microRNA-181 determines response to treatment with azacitidine and predicts survival in elderly patients with acute myeloid leukemia. Oncol. Lett..

[179] Su R., Lin H.S., Zhang X.H., Yin X.L., Ning H.M., Liu B., Zhai P.F., Gong J.N., Shen C., Song L. (2015). MiR-181 family: Regulators of myeloid differentiation and acute myeloid leukemia as well as potential therapeutic targets. Oncogene.

[180] Gao X., Fan S., Zhang X. (2022). MiR-1306-5p promotes cell proliferation and inhibits cell apoptosis in acute myeloid leukemia by downregulating PHF6 expression. Leuk. Res..

[181] Jiang X., Hu C., Arnovitz S., Bugno J., Yu M., Zuo Z., Chen P., Huang H., Ulrich B., Gurbuxani S. (2016). miR-22 has a potent anti-tumor role with therapeutic potential in acute myeloid leukemia. Nat. Commun..

[182] Shen C., Chen M.T., Zhang X.H., Yin X.L., Ning H.M., Su R., Lin H.S., Song L., Wang F., Ma Y.N. (2016). The PU.1-Modulated MicroRNA-22 Is a Regulator of Monocyte/Macrophage Differentiation and Acute Myeloid Leukemia. PLoS Genet..

[183] Qu H., Zheng G., Cheng S., Xie W., Liu X., Tao Y., Xie B. (2020). Serum miR-22 is a novel prognostic marker for acute myeloid leukemia. J. Clin. Lab. Anal..

[184] Yao H., Sun P., Duan M., Lin L., Pan Y., Wu C., Fu X., Wang H., Guo L., Jin T. (2017). microRNA-22 can regulate expression of the long non-coding RNA MEG3 in acute myeloid leukemia. Oncotarget.

[185] Gu Y., Si J., Xiao X., Tian Y., Yang S. (2017). miR-92a Inhibits Proliferation and Induces Apoptosis by Regulating Methylenetetrahydrofolate Dehydrogenase 2 (MTHFD2) Expression in Acute Myeloid Leukemia. Oncol. Res..

[186] Su X.Y., Zhao Q., Ke J.M., Wu D.H., Zhu X., Lin J., Deng Z.Q. (2020). Circ_0002232 Acts as a Potential Biomarker for AML and Reveals a Potential ceRNA Network of Circ_0002232/miR-92a-3p/PTEN. Cancer Manag. Res..

[187] Saadi M.I., Arandi N., Yaghobi R., Azarpira N., Geramizadeh B., Ramzi M. (2018). Up-Regulation of the MiR-92a and miR-181a in Patients with Acute Myeloid Leukemia and their Inhibition with Locked Nucleic acid (LNA)-antimiRNA.; Introducing c-Kit as a New Target Gene. Int. J. Hematol. Oncol..

[188] Rashed R.A., Hassan N.M., Hussein M.M. (2020). MicroRNA-92a as a marker of treatment response and survival in adult acute myeloid leukemia patients. Leuk. Lymphoma.

[189] Qi X., Zhang Y. (2022). MicroRNA-199a deficiency relates to higher bone marrow blasts, poor risk stratification and worse prognostication in pediatric acute myeloid leukemia patients. Pediatr. Hematol. Oncol..

[190] Li Y., Zhang G., Wu B., Yang W., Liu Z. (2019). miR-199a-5p Represses Protective Autophagy and Overcomes Chemoresistance by Directly Targeting DRAM1 in Acute Myeloid Leukemia. J. Oncol..

[191] Ellson I., Martorell-Marugán J., Carmona-Sáez P., Ramos-Mejia V. (2024). MiRNA expression as outcome predictor in pediatric AML: Systematic evaluation of a new model. npj Genom. Med..

[192] Favreau A.J., McGlauflin R.E., Duarte C.W., Sathyanarayana P. (2016). miR-199b, a novel tumor suppressor miRNA in acute myeloid leukemia with prognostic implications. Exp. Hematol. Oncol..

[193] Alemdehy M.F., Haanstra J.R., de Looper H.W., van Strien P.M., Verhagen-Oldenampsen J., Caljouw Y., Sanders M.A., Hoogenboezem R., de Ru A.H., Janssen G.M. (2015). ICL-induced miR139-3p and miR199a-3p have opposite roles in hematopoietic cell expansion and leukemic transformation. Blood.

[194] Yuan Y., Tan S., Wang H., Zhu J., Li J., Zhang P., Wang M., Zhang F. (2023). Mesenchymal Stem Cell-Derived Exosomal miRNA-222-3p Increases Th1/Th2 Ratio and Promotes Apoptosis of Acute Myeloid Leukemia Cells. Anal. Cell. Pathol..

[195] Pei H.Z., Peng Z., Zhuang X., Wang X., Lu B., Guo Y., Zhao Y., Zhang D., Xiao Y., Gao T. (2023). miR-221/222 induce instability of p53 By downregulating deubiquitinase YOD1 in acute myeloid leukemia. Cell Death Discov..

[196] Liu Z., Zhong L., Dan W., Chu X., Liu C., Luo X., Zhang Z., Lu Y., Wan P., Wang X. (2022). miRNA-222-3p enhances the proliferation and suppresses the apoptosis of acute myeloid leukemia cells by targeting Axin2 and modulating the Wnt/β-catenin pathway. Biochem. Biophys. Res. Commun..

[197] Pavlovic D., Tosic N., Zukic B., Pravdic Z., Vukovic N.S., Pavlovic S., Gasic V. (2021). Expression Profiles of Long Non-Coding RNA GAS5 and MicroRNA-222 in Younger AML Patients. Diagnostics.

[198] Xu D., Jiang J., He G., Zhou H., Ji C. (2023). KMT2A is targeted by miR-361-3p and modulates leukemia cell’s abilities to proliferate, migrate and invade. Hematology.

[199] Liu S., Xu H., Li Z. (2023). Linoleic acid derivatives target miR-361-3p/BTG2 to confer anticancer effects in acute myeloid leukemia. J. Biochem. Mol. Toxicol..

[200] Johnson D.T., Davis A.G., Zhou J.H., Ball E.D., Zhang D.E. (2021). MicroRNA let-7b downregulates AML1-ETO oncogene expression in t(8;21) AML by targeting its 3′UTR. Exp. Hematol. Oncol..

[201] Fazi F., Racanicchi S., Zardo G., Starnes L.M., Mancini M., Travaglini L., Diverio D., Ammatuna E., Cimino G., Lo-Coco F. (2007). Epigenetic silencing of the myelopoiesis regulator microRNA-223 by the AML1/ETO oncoprotein. Cancer Cell.

[202] Fu L., Shi J., Liu A., Zhou L., Jiang M., Fu H., Xu K., Li D., Deng A., Zhang Q. (2017). A minicircuitry of microRNA-9-1 and RUNX1-RUNX1T1 contributes to leukemogenesis in t(8;21) acute myeloid leukemia. Int. J. Cancer.

[203] Li Z., Chen P., Su R., Li Y., Hu C., Wang Y., Arnovitz S., He M., Gurbuxani S., Zuo Z. (2015). Overexpression and knockout of miR-126 both promote leukemogenesis. Blood.

[204] Krivdova G., Voisin V., Schoof E.M., Marhon S.A., Murison A., McLeod J.L., Gabra M.M., Zeng A.G.X., Aigner S., Yee B.A. (2022). Identification of the global miR-130a targetome reveals a role for TBL1XR1 in hematopoietic stem cell self-renewal and t(8;21) AML. Cell Rep..

[205] Li Y., Ning Q., Shi J., Chen Y., Jiang M., Gao L., Huang W., Jing Y., Huang S., Liu A. (2017). A novel epigenetic AML1-ETO/THAP10/miR-383 mini-circuitry contributes to t(8;21) leukaemogenesis. EMBO Mol. Med..

[206] Yuan Z., Zhong L., Liu D., Yao J., Liu J., Zhong P., Yao S., Zhao Y., Li L., Chen M. (2019). MiR-15b regulates cell differentiation and survival by targeting CCNE1 in APL cell lines. Cell. Signal..

[207] Guo B., Qin R., Chen J.J., Pan W., Lu X.C. (2022). MicroRNA-125b Accelerates and Promotes PML-RARa-driven Murine Acute Promyelocytic Leukemia. Biomed. Environ. Sci..

[208] Liu D., Zhong L., Yuan Z., Yao J., Zhong P., Liu J., Yao S., Zhao Y., Liu L., Chen M. (2019). miR-382-5p modulates the ATRA-induced differentiation of acute promyelocytic leukemia by targeting tumor suppressor PTEN. Cell. Signal..

[209] Ovcharenko D., Stölzel F., Poitz D., Fierro F., Schaich M., Neubauer A., Kelnar K., Davison T., Müller-Tidow C., Thiede C. (2011). miR-10a overexpression is associated with NPM1 mutations and MDM4 downregulation in intermediate-risk acute myeloid leukemia. Exp. Hematol..

[210] Gadewal N., Kumar R., Aher S., Gardane A., Gaur T., Varma A.K., Khattry N., Hasan S.K. (2020). miRNA-mRNA Profiling Reveals Prognostic Impact of SMC1A Expression in Acute Myeloid Leukemia. Oncol. Res..

[211] Rücker F.G., Russ A.C., Cocciardi S., Kett H., Schlenk R.F., Botzenhardt U., Langer C., Krauter J., Fröhling S., Schlegelberger B. (2013). Altered miRNA and gene expression in acute myeloid leukemia with complex karyotype identify networks of prognostic relevance. Leukemia.

[212] Shahzad M., Amin M.K., Daver N.G., Shah M.V., Hiwase D., Arber D.A., Kharfan-Dabaja M.A., Badar T. (2024). What have we learned about TP53-mutated acute myeloid leukemia?. Blood Cancer J..

[213] Chen Y., Chen S., Lu J., Yuan D., He L., Qin P., Tan H., Xu L. (2021). MicroRNA-363-3p promote the development of acute myeloid leukemia with RUNX1 mutation by targeting SPRYD4 and FNDC3B. Medicine.

[214] Barreyro L., Sampson A.M., Hueneman K., Choi K., Christie S., Ramesh V., Wyder M., Wang D., Pujato M., Greis K.D. (2024). Dysregulated innate immune signaling cooperates with RUNX1 mutations to transform an MDS-like disease to AML. iScience.

